# Interaction of the endogenous antibody response with activating FcγRs enhance control of Mayaro virus through monocytes

**DOI:** 10.1371/journal.ppat.1012944

**Published:** 2025-02-24

**Authors:** Megan M. Dunagan, Nathânia Dábilla, Colton McNinch, Jason M. Brenchley, Patrick T. Dolan, Julie M. Fox

**Affiliations:** 1 Emerging Virus Immunity Unit, Laboratory of Viral Diseases, National Institute of Allergy and Infectious Diseases, National Institutes of Health, Bethesda, Maryland, United States of America; 2 Quantitative Virology and Evolution Unit, Laboratory of Viral Diseases, National Institute of Allergy and Infectious Diseases, National Institutes of Health, Bethesda, Maryland, United States of America; 3 Bioinformatics and Computational Bioscience Branch, National Institute of Allergy and Infectious Diseases, National Institutes of Health, Rockville, Maryland, United States of America; 4 Barrier Immunity Section, Laboratory of Viral Diseases, National Institute of Allergy and Infectious Diseases, National Institutes of Health, Bethesda, Maryland, United States of America; University of North Carolina at Chapel Hill, UNITED STATES OF AMERICA

## Abstract

Mayaro virus (MAYV) is an emerging arbovirus. Previous studies have shown antibody Fc effector functions are critical for optimal monoclonal antibody-mediated protection against alphaviruses; however, the requirement of Fc gamma receptors (FcγRs) for protection during natural infection has not been evaluated. Here, we showed mice lacking activating FcγRs (FcRγ^−/−^) developed prolonged clinical disease with increased MAYV in joint-associated tissues. Viral reduction was associated with anti-MAYV cell surface binding antibodies rather than neutralizing antibodies. Lack of Fc-FcγR engagement increased the number of monocytes present in the joint-associated tissue through chronic timepoints. Single-cell RNA sequencing showed elevated levels of pro-inflammatory monocytes in joint-associated tissue with increased MAYV RNA present in FcRγ^−/−^ monocytes and macrophages. Transfer of FcRγ^−/−^ monocytes into wild type animals was sufficient to increase virus in joint-associated tissue. Overall, this study suggests that engagement of antibody Fc with activating FcγRs promotes protective responses during MAYV infection and prevents a pro-viral role for monocytes.

## Author summary

Arthritogenic alphaviruses, including Mayaro virus (MAYV), are mosquito-transmitted viruses of global concern. Antibodies are important for clearance of infectious virus; however, the specific antibody activities (neutralization versus Fc effector functions) required for optimal protection during alphavirus infection have not been evaluated. Fc effector functions can enhance viral clearance and alter the immune response through engagement of the antibody Fc domain with host receptors, such as Fc gamma receptors (FcγRs). Here, we infected mice that lack activating FcγRs (FcRγ^−/−^) with MAYV to determine the importance of Fc-FcγR interactions during natural alphavirus infection. In our study, FcRγ^−/−^ mice showed prolonged disease and infection in the joint-associated tissue. Reduction of MAYV in the joint-associated tissue was more dependent on Fc-FcγR engagement rather than neutralizing antibodies. FcRγ^−/−^ mice had more monocytes present during disease resolution time points and these cells harbored more MAYV RNA as compared to wild type (WT) mice. The FcRγ^−/−^ monocytes failed to produce appreciable levels of infectious MAYV; however, transfer of these cells into WT mice increased MAYV burden within the joint-associated tissue. This study suggests that engagement of antibody Fc with activating FcγRs promotes a protective response during MAYV infection and prevents monocytes from driving a prolonged infection.

## Introduction

Alphaviruses are transmitted by mosquitoes and have caused explosive outbreaks worldwide [1]. Arthritogenic alphaviruses, including chikungunya virus (CHIKV), Ross River virus (RRV), and Mayaro virus (MAYV), can cause fever, myalgia, and arthralgia. Up to 50% of infected individuals can develop polyarthralgia lasting months to years following initial infection, with rare cases showing neurological complications or even death [2–5]. Since its identification in 1954, MAYV has caused occasional outbreaks in rural areas of Central and South America as well as the Caribbean [6]. While MAYV is primarily transmitted by forest dwelling *Haemagogus spp.* mosquitoes, *Aedes aegypti* have been shown experimentally to be competent vectors for MAYV, highlighting the potential for MAYV to spread into more populated urban regions [7]. Despite these risks MAYV remains understudied, as cases are largely underreported or misdiagnosed [8–10]. Understanding the aspects of immunity that contribute to pathogenesis and disease resolution will inform the development of vaccines and therapeutics.

Alphaviruses are enveloped, positive-sense RNA viruses with a genome encoding four nonstructural proteins (nsP1- nsP4) and six structural proteins (capsid, E3, E2, 6K, TF, and E1) [11]. Following viral replication, trimers of p62 (E3 and E2) and E1 are assembled and trafficked to the cell surface for subsequent budding of progeny virions [12]. During transport through the trans-Golgi network, furin-like proteases cleave E3 to produce the mature E2-E1 heterodimer [13]. The E2 and E1 surface glycoproteins mediate viral attachment and fusion, respectively, and have been characterized broadly as targets of the antibody responses following alphavirus infection [14–18]. Anti-alphavirus monoclonal antibodies (mAbs) have been shown to block multiple stages in the viral life cycle including attachment, entry, fusion, and egress [19–21]. Furthermore, antibodies can bind to the E2 and E1 proteins present on the infected cell surface, in addition to free virions, and mediate enhanced clearance and immune modulation through Fc interaction with host proteins [*e.g*., Fc gamma receptors (FcγRs) and the complement component, C1q] [22–24].

Mouse models of alphavirus disease recapitulate key aspects of human infection. MAYV infection in an immunocompetent [C57BL/6 wild type (WT)] mouse model results in broad viral dissemination, high viral titers, symmetric joint swelling, and a robust innate and adaptive immune response [18,25]. Similar to mouse models of CHIKV and RRV, antibodies are necessary to clear circulating infectious MAYV; T and B cell deficient (RAG^−/−^) mice survive MAYV infection with sustained viremia, and administration of cross-reactive alphavirus immune serum suppresses MAYV viremia to undetectable levels for a short duration [26–28]. Previous studies evaluating mAb efficacy against CHIKV or MAYV showed a requirement for Fc-mediated activity for optimal protection [18,19,23,29]. For MAYV, the necessity of Fc effector functions for protection was independent of *in vitro* neutralization potency [18,30]. These studies highlight the importance of Fc-FcγR interactions for mAb-mediated protection during alphavirus infection. However, these studies administered mAbs either before or within a few days of infection, which is prior to the generation of an endogenous humoral response. As such, the contribution of Fc-FcγR interactions during primary MAYV infection remains unclear.

Here, we evaluated the role of Fc-FcγR interactions for disease resolution during a primary MAYV infection using mice that lack the Fc receptor common gamma chain (FcRγ^−/−^) and thus do not express activating FcγRs [31]. Mice lacking activating FcγRs showed prolonged foot swelling and increased viral RNA and infectious virus during disease resolution, despite having similar levels of binding and neutralizing antibodies. Infection of B cell-depleted or mice lacking mature B cells demonstrated that FcγR interaction with anti-MAYV reactive antibodies, rather than the presence of neutralizing antibodies, mediated MAYV reduction in joint-associated tissue. FcRγ^−/−^ mice had increased infiltration of immune cells into the joint-associated tissue during acute disease with an altered proportion of monocytes to macrophages which persisted to a chronic time point. Analysis of the myeloid cell populations by single-cell RNA sequencing showed increased viral RNA in monocytes and macrophage clusters, which corresponded with enrichment of pathways associated with type I IFN signaling, antiviral response, and cellular stress response. Adoptive transfer of FcRγ^−/−^ monocytes was sufficient to increase viral burden in WT mice. Overall, these studies indicate that Fc-FcγR interactions are necessary to prevent prolonged MAYV infection and disease and activating FcγR engagement on monocytes may suppress a pro-viral response.

## Results

### Activating FcγRs enhance disease resolution and viral control during MAYV infection

**Fig 1:**
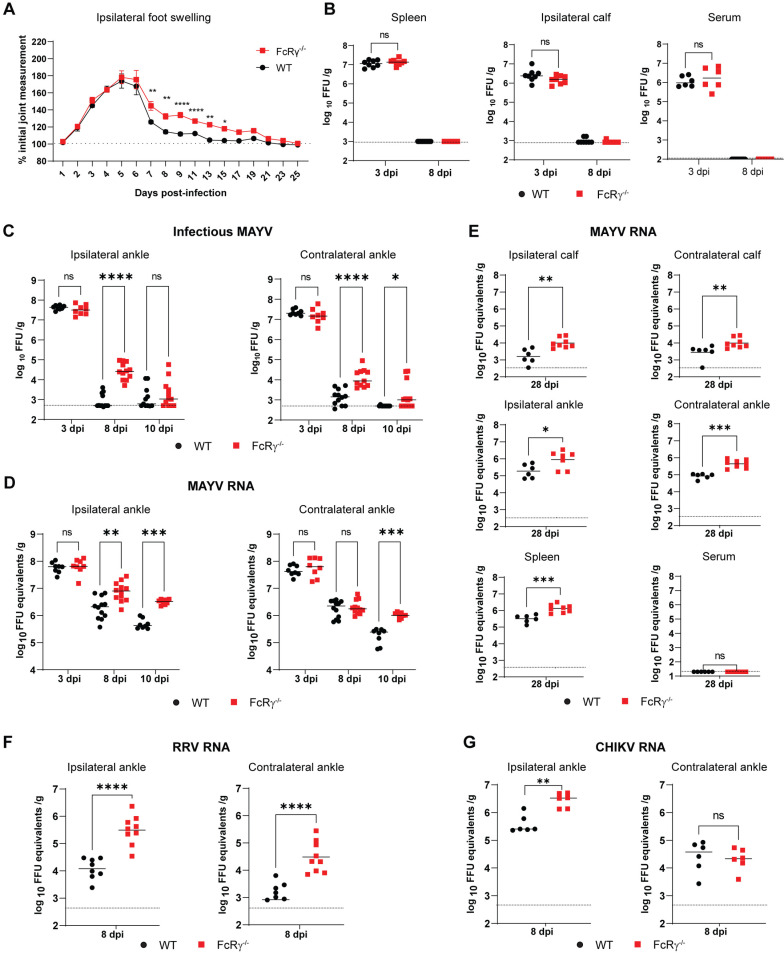
Prolonged foot swelling and viral burden in joint-associated tissue of FcRγ−/− mice following MAYV infection. Four-week-old WT or FcRγ^−/−^ C57BL/6N mice were infected subcutaneous in the rear footpad with 10^3^ focus forming units (FFU) of (A–E) MAYV, (F) RRV, or (G) CHIKV. (A) Swelling of the ipsilateral foot was measured prior to infection and for 25 dpi (n = 8 per group, 2 independent experiments). Graphs show mean ± SEM. Statistical significance was determined using a two-way ANOVA with repeated measures and a Sidak’s post-test at each time point (*, *P* < 0.05; **, *P* < 0.01; ****, *P <* 0.0001). (B–G) Indicated tissues were harvested at 3, 8, or 10 dpi and titrated for (B, C) infectious virus by focus forming assay (FFA) or (D–G) viral RNA by qRT-PCR with virus specific primers and probe (n = 8 to 12 per group; 2 to 3 independent experiments). Statistical significance was determined by a Mann-Whitney test. (*, ****P**** <0.05; **, ****P**** < 0.01; ***, ****P**** < 0.001; ****, ****P**** < 0.0001; ns = not significant.) Bars indicate the median value and dotted lines indicate the limit of detection for the assay.

In the immunocompetent mouse model of MAYV-induced musculoskeletal disease, MAYV inoculation in the footpad results in swelling of both the infected (ipsilateral) and contralateral foot through 8 days post-infection (dpi), with peaked planar edema between 5 to 6 dpi in the ipsilateral foot and infectious virus measurable through 10 dpi [25]. To assess the contribution of activating FcγRs during MAYV infection, we inoculated four-week-old C57BL/6N WT or FcRγ^−/−^ mice subcutaneously in the rear footpad with 10^3^ focus forming units (FFU) of MAYV and measured foot swelling through 25 dpi. As expected, swelling of the ipsilateral and contralateral feet peaked between 5 to 6 dpi and substantially decreased by 8 dpi in WT mice (Figs 1A and S1A). While foot swelling still peaked at 5 to 6 dpi in FcRγ^−/−^ mice, with no difference in the overall magnitude of swelling, the absence of activating FcγRs lead to prolonged swelling until 15 dpi in the ipsilateral foot (Fig 1A) and a trend toward increased swelling, although not statistically significant, in the contralateral foot (S1A Fig).

Following infection, MAYV rapidly disseminates causing high viremia and viral burden in skeletal muscles, spleen, and joint-associated tissues by 3 dpi. Similar levels of infectious virus were observed at 3 dpi in spleen, gastrocnemius (calf) muscle, and serum of WT and FcRγ^−/−^ mice (Fig 1B). By 8 dpi, infectious virus was not detectable in these tissues but there was a significant increase in viral RNA in the spleens of FcRγ^−/−^ mice (S1B Fig). We next quantified infectious MAYV and MAYV RNA from joint-associated tissues. While there were similar viral loads at 3 dpi, FcRγ^−/−^ mice showed prolonged infectious virus and viral RNA in the ipsilateral and contralateral ankles at 8 and/or 10 dpi compared to WT mice (Fig 1C and 1D). The delay in viral reduction was maintained at a chronic time point (28 dpi), with FcRγ^−/−^ mice having significantly more MAYV RNA in the ankles, calves, and spleen compared to WT mice (Fig 1E). These data suggest that prolonged MAYV infection resulting from the lack of activating FcγRs are most notable in joint-associated tissue during acute infection but can also persist at the RNA level in other tissues during chronic time points.

To determine if FcγR-mediated reduction in viral burden applied more broadly to arthritogenic alphaviruses, we infected WT or FcRγ^−/−^ mice with either RRV or CHIKV and quantified viral RNA from the ankles at 8 dpi (Fig 1F and 1G). Viral RNA levels were increased in FcRγ^−/−^ mice compared to WT mice in the ipsilateral and contralateral ankles following RRV infection and only in the ipsilateral ankle following CHIKV infection suggesting that activating FcγRs are more broadly required for optimal reduction of viral RNA during primary alphavirus infection.

### FcγR interaction with cell surface binding antibodies is necessary for a reduction in MAYV burden

**Fig 2:**
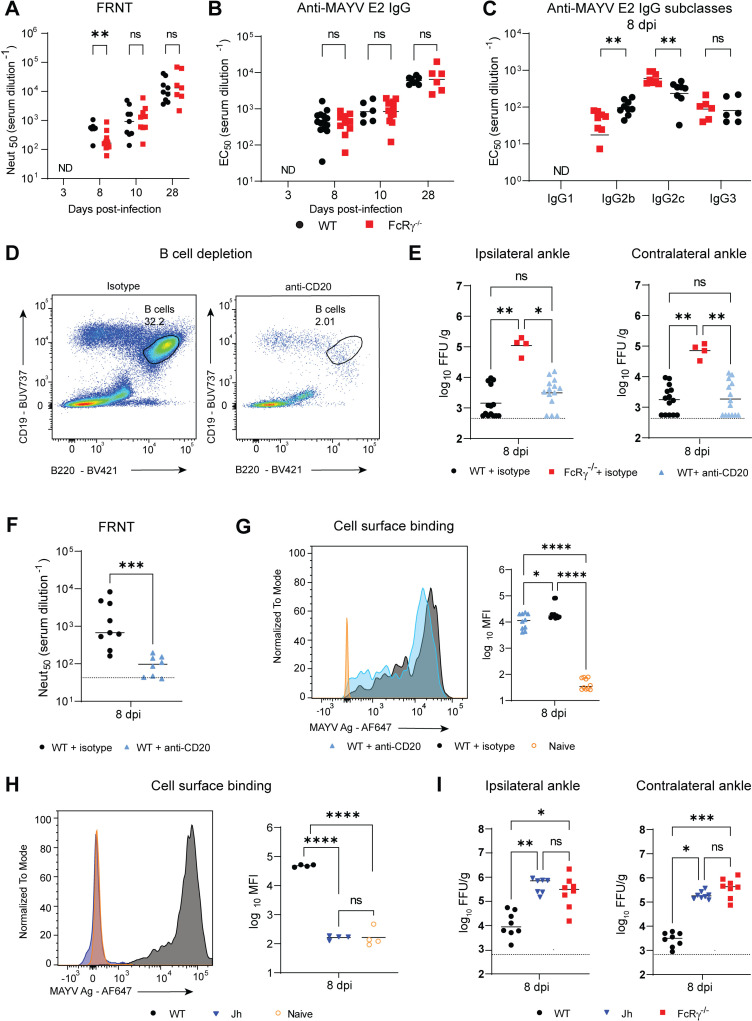
Interaction of cell surface binding anti-MAYV antibodies with activating FcγRs mediate MAYV reduction in joint-associated tissues. (A-C) Four-week-old WT or FcRγ^−/−^ C57BL/6N mice were infected subcutaneous in the rear footpad with 10^3^ FFU of MAYV. Serum was collected at indicated time points. Serial dilutions of serum were used to determine (A) Neut_50_ values for MAYV neutralization by focus reduction neutralization test (FRNT) and EC_50_ values of (B) anti-MAYV E2-specific IgG or (C) IgG subclasses by ELISA (n = 8 to 15 per group; 2 to 3 independent experiments). (D-G) B cells were depleted using anti-CD20 administered on -1 and 4 dpi (500 µg/dose) and control mice received a non-depleting isotype control antibody (n = 4 to 8 per group; 2 independent experiments). (D) PBMCs were collected at 8 dpi to confirm B cell (CD19^+^B220^+^) depletion by flow cytometry. (E) Infectious virus was titrated from the ipsilateral and contralateral ankles by FFA at 8 dpi. (F) Neut_50_ values of serum tested for MAYV neutralization by FRNT. (G) Serum antibodies binding to the surface of live MAYV-infected Vero cells was evaluated by flow cytometry. (H-I) Jh, WT, or FcRγ^−/−^ mice were infected with MAYV and tissues were collected at 8 dpi. (H) Cell surface MAYV-binding antibodies were quantified from the serum and (I) infectious virus was titrated from the ankles (n = 4 to 8; 2 independent experiments). Bars indicate the median values. Statistical significance was determined by a Mann-Whitney test (A, B, C, and F), one-way ANOVA with Tukey’s post**-**test (G, H) or Kruskal-Wallis test with a Dunn’s post-test (E, I). *, ****P**** <0.05; **, ****P**** < 0.01; ***, ****P**** < 0.001; ****, ****P**** < 0.0001; ns = not significant; ND = not detected.

Previous studies have highlighted the importance of antibodies for the clearance of infectious alphaviruses [19,30,32]. Despite the lack of activating FcγRs on B cells, variations in the tissue microenvironment could impact the anti-MAYV antibody response following infection in the FcRγ^−/−^ mice [31,33]. To determine if the FcRγ^−/−^ mice had an altered antibody response, we characterized antibody quality and quantity against MAYV surface glycoproteins and focused on time points associated with increased swelling and infectious virus in FcRγ^−/−^ mice (Fig 1A and 1C). As expected, no anti-MAYV antibodies were detectable in circulation at 3 dpi (Fig 2A and 2B). At 8 dpi, FcRγ^−/−^ mice had significantly lower levels of neutralizing antibodies but equivalent antibody neutralization titers between the groups by 10 dpi through 28 dpi (Fig 2A). This variation in neutralization at 8 dpi could not be explained by differences in avidity (S2A Fig) nor by differences in the quantity of total IgG or IgM against MAYV or, specifically, to the E2 surface glycoprotein (Figs 2B and S2B). Interestingly, there were transient differences in the IgG subclasses with FcRγ^−/−^ mice having increased IgG2c and decreased IgG2b when compared to WT mice at 8 dpi (Fig 2C). Importantly, IgG2c was still the dominant IgG subclass in both groups and this shift did not affect antibody dependent cellular cytotoxicity (ADCC) as tested using an *in vitro* assay (S2D Fig). By 28 dpi, equivalent levels of each IgG subclass were present in the WT and FcRγ^−/−^ mice (S2C Fig). These results indicate that, while there is a delay in the early generation of neutralizing antibody, there is no dramatic defect in antibody response in FcRγ^−/−^ mice.

To begin to evaluate the requirement of antibody interaction with the activating FcγRs for viral control, we depleted B cells using an anti-CD20 antibody, as previously described [34]. Since the mechanism of anti-CD20 is dependent on activating FcγRs, we could not deplete B cells in the FcRγ^−/−^ mice. Instead, WT mice administered two doses of an anti-CD20 antibody or isotype control (500 µg; 0 and 4 dpi) were compared to FcRγ^−/−^ mice administered an isotype control. Consistent with previous data, anti-CD20 antibody treatment dramatically decreased double positive B220^+^CD19^+^ B cells from the blood of infected mice at 8 dpi (Fig 2D). Surprisingly, B cell depletion failed to increase infectious virus burden to the level observed in FcRγ^−/−^ mice. Instead, viral burden recapitulated WT mice administered an isotype control in both ankles (Fig 2E). As a secondary measure of B cell depletion, we quantified levels of neutralizing and cell surface binding antibodies in the serum at 8 dpi. While B cell depletion significantly reduced the level of neutralizing antibodies (Fig 2F), a significant amount of MAYV-binding antibody remained, with, on average, only a 2.5-fold decrease in the staining intensity of IgG that bound to the surface of MAYV-infected Vero cells compared to the isotype-treated mice (Fig 2G). These results show there was still significant anti-MAYV antibody generated despite anti-CD20 depletion, which is most likely due to a combination of incomplete B cell depletion from tissues and recovery of B cell populations following depletion [35]. Of note, the substantial reduction in neutralizing antibodies only marginally impacted the reduction of infectious virus from joint-associated tissue indicating a more dominant role for antibodies that bind to the surface of infected cells for MAYV clearance.

To fully address the contribution of soluble antibody to MAYV reduction in joint-associated tissue, we infected Jh (C57BL/6N) mice with MAYV and quantified infectious virus in the ankles at 8 dpi. Jh mice lack the J segment of the Ig heavy chain, a mutation that stalls B cell development at the precursor stage preventing the production of antibodies [36]. Indeed, serum collected from Jh mice at 8 dpi did not contain IgG that bound to the surface of MAYV-infected Vero cells (Fig 2H). Jh mice failed to efficiently reduce infectious virus from the ankles as compared to the WT mice (Fig 2I). Interestingly, the viral burden in ankles of Jh mice was similar to the viral load in FcRγ^−/−^ mice at 8 dpi even though FcRγ^−/−^ mice still have neutralizing antibodies present (Fig 2A and 2I). Taken together, these results suggest that antibody-mediated reduction of MAYV in joint-associated tissues depend more on interaction of activating FcγRs with, presumably, the Fc region of antibodies rather than the presence of neutralizing antibodies.

### Prolonged recruitment of immune cells to the site of infection in the absence of activating FcγRs

**Fig 3:**
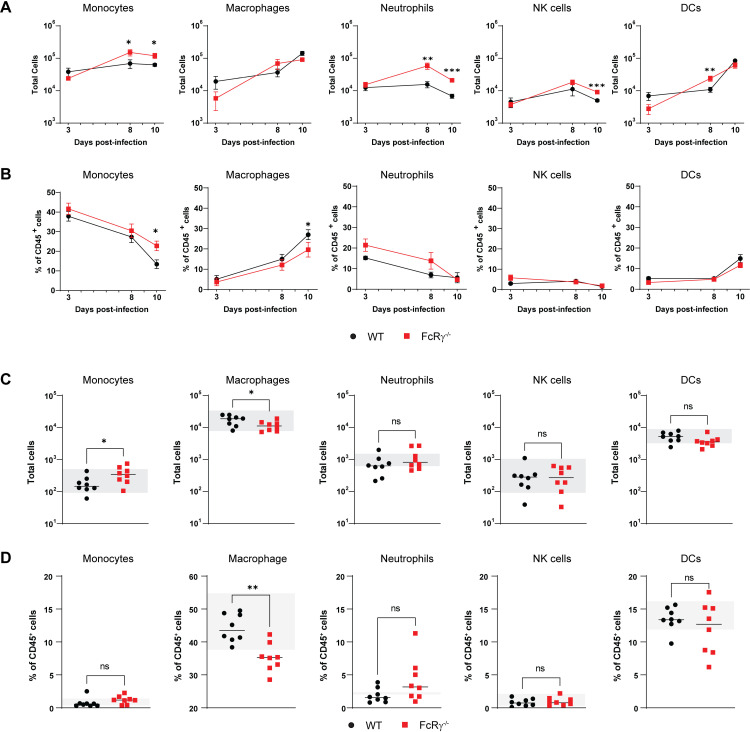
Lack of activating FcγRs alters flux of immune cells in the ipsilateral foot. Four-week-old WT or FcRγ^−/−^ C57BL/6N mice were infected subcutaneous in the rear footpad with 10^3^ FFU of MAYV. Single cell suspensions were isolated from the ipsilateral foot and proximal skin at (A-B) 3, 8, and 10 dpi or (C-D) 28 dpi and stained for monocytes (Ly6C^hi^), macrophages (Ly6C^mid-lo^F4/80^+^), neutrophils (Ly6G^+^), NK cells (NK1.1^+^), and dendritic cells (DCs; CD11b^-^CD11c^+^ MHCII^+^) and analyzed by flow cytometry to determine the (**A, C**) total numbers of viable cells or (B, D) percentage of CD45^+^ cells (n = 5 to 8 per group; 3 independent experiments). (C, D) The gray bar represents the range of (**C**) total cells and (**D**) percentage of CD45^+^ cells from WT and FcRγ^−/−^ naïve mice. Graphs show mean ± SEM. Statistical significance was determined using a Mann-Whitney test at individual time points. *, ****P**** <0.05; **, ****P**** < 0.01; ***, ****P**** < 0.001; ****, ****P**** < 0.0001; ns = not significant.

Recruitment of inflammatory immune cells has been implicated in both protection as well as immunopathology following alphavirus infection. While little is known about cellular contributors to disease during MAYV infection, infiltrating CD4^+^ T cells, and monocytes/macrophage have been shown to contribute to disease in CHIKV models and FcγR engagement with anti-CHIKV mAbs was shown to alter immune cell infiltration [22,37]. To determine if FcγR engagement with the endogenous humoral response alters immune cell responses, the ipsilateral foot was harvested from MAYV-infected WT or FcRγ^−/−^ mice at 3, 8, 10, or 28 dpi. Following digestion, single cell suspensions were stained and analyzed by flow cytometry (S3A Fig). In contrast to WT mice, FcRγ^−/−^ mice had an altered flux of several immune cell populations across this time course. Of the cellular subsets that normally express activating FcγRs in mice [33], FcRγ^−/−^ mice had increased numbers of neutrophils, NK cells, and Ly6C^hi^ monocytes between 8-10 dpi (Fig 3A), with WT mice having proportionally more Ly6C^mid-low^F4/80^+^ macrophages and fewer Ly6C^hi^ monocytes compared to FcRγ^−/−^ mice at 10 dpi (Fig 3B). While most immune cell populations had returned to within naïve ranges at 28 dpi, there were significantly more monocytes with a corresponding reduction in macrophage in FcRγ^−/−^ mice compared to WT mice (Fig 3C). The proportion of macrophages in the FcRγ^−/−^ tissue was even below naïve levels, suggesting a defect in the return of cellular homeostasis in the absence of activating FcγRs (Fig 3D). Additionally, FcRγ^−/−^ mice had increased numbers of CD8^+^ T cells and B cells, but not CD4^+^ T cells, at both early and late timepoints following MAYV infection (S3B and S3C Fig). Taken together, these results show that the lack of activating FcγR engagement leads to an increase in monocytes, neutrophils, NK cells, B cells, and CD8^+^ T cells in the joint-associated tissue during acute disease resolution which may explain the prolonged disease observed in FcRγ^−/−^ mice.

### Increased MAYV RNA in monocyte and macrophages without activating FcγRs

The shift in key immune cell populations at both early and chronic time points in the FcRγ^−/−^ mice suggest a differential response between these cellular populations. To characterize the cellular responses that could be driving either disease resolution or prolongment, we performed single-cell RNA sequencing on the ipsilateral foot from naïve or MAYV-infected WT or FcRγ^−/−^ mice at 10 dpi to evaluate the transcriptomic profile of immune cells as well as identify the presence of MAYV RNA. Single cell suspensions were stained, and cells were sorted based on CD45 expression. CD45^+^ cells and CD45^-^ cells were subjected to micro-fluidics-based single-cell RNA sequencing with the addition of MAYV-specific primers. More than 2,000 cells were collected from each group and > 100,000 RNA reads were analyzed per cell, with CD45^+^ immune cell subsets identified based on key markers and expert curation (S4A–S4C Fig) [38–62].

**Fig 4:**
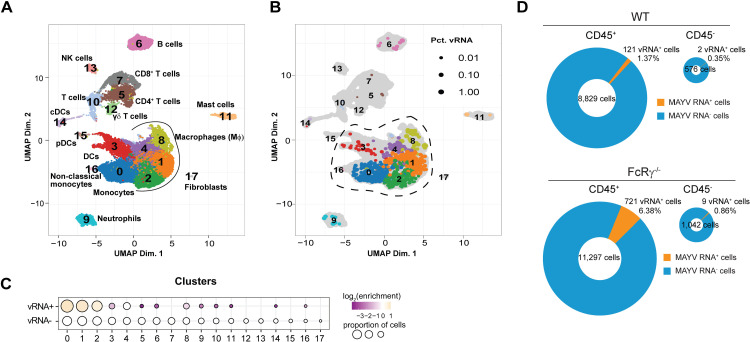
Increased MAYV RNA in myeloid cells lacking activating FcγRs. Four-week-old WT or FcRγ^−/−^ C57BL/6N were infected subcutaneous in the rear footpad with 10^3^ FFU of MAYV or mock infected with diluent. At 10 dpi, the ipsilateral foot and surrounding skin was enzymatically digested into a single cell suspension and stained for CD45. Viable CD45^+^ and CD45^-^ cells were sorted then subjected to microfluidic-based single cell RNA sequencing with the addition of MAYV-specific primers. Over 2000 cells were collected per group of the CD45^+^ cells and from 300 to 700 cells for CD45^-^ cells, with >100,000 RNA reads per cell. (A) UMAP shows the integrated cell clusters from all groups of the CD45^+^ cells. (B) Cells containing MAYV RNA are identified based on the cluster color in (**A**) with the size of the dot represents the percentage of viral RNA in the cells. The dotted line indicates cells included in additional subcluster analysis (Fig 5). (C) The proportion of cells, across each integrated cluster segregated based on the presence of MAYV RNA, indicating the log_2_(enrichment) of each cluster between viral RNA positive and negative cells. (D) Percentage of either CD45^+^ or CD45^-^ cells that were positive for MAYV RNA.

Integrated analysis of the CD45^+^ cells from all samples showed the presence of distinct immune cell clusters encompassing similar cellular populations identified in our flow cytometry analysis (Fig 4A). These populations included NK cells, B cells, CD8^+^ and CD4^+^ T cells, macrophages, monocytes, neutrophils, and dendritic cells (DCs) with the addition of mast cells and γδ T cells. The proportion and enrichment of each cluster was consistent between replicates from the infected groups highlighting the reproducibility of the analysis (S4D Fig). We next evaluated the distribution of MAYV RNA within the integrated data set. There was a clear enrichment of viral RNA in myeloid cell clusters including Clusters 0, 1, 2, 3, 4, and 8 (Fig 4B and 4C). Interestingly, MAYV RNA was predominantly observed in the CD45^+^ cell populations at this timepoint, with elevated viral RNA in FcRγ^−/−^ CD45^+^ cells compared to the WT CD45^+^ cells and minimal differences detected in viral RNA quantity between WT and FcRγ^−/−^ CD45^-^ cells (Figs 4D and S4E).

While CD8^+^ T cells were increased in FcRγ^−/−^ mice (S3C Fig), this cluster (Cluster 7) did not contain viral RNA. However, the CD8^+^ T cells may be compensating in the FcRγ^−/−^ mice. To determine differences in the CD8^+^ T cell (Cluster 7) response, we performed ingenuity pathway analysis (IPA) on differentially expressed genes (DEGs) enriched in the FcRγ^−/−^ mice compared to WT mice. DEGs and pathway analysis identified protein translation and cellular stress response pathways positively enriched (z score > 0) in FcRγ^−/−^ mice compared to WT mice as well as negative enrichment (z score < 0) for the role of PKR in interferon induction and antiviral responses (S5A–S5C Fig). Importantly, there were no significant enrichment of genes associated with T cell killing, such as granzymes (*Gzma, Gzma, Gzmk*), perforin (*Prf1*), or IFNγ (*Ifng*), suggesting there is not enhanced CD8-mediated killing in the FcRγ^−/−^ mice. Since CD8^+^ T cells have been shown to minimally contribute to viral control, specifically in joint-associated tissue, or disease resolution following alphavirus infection we did not further analyze these cells [63,64].

**Fig 5:**
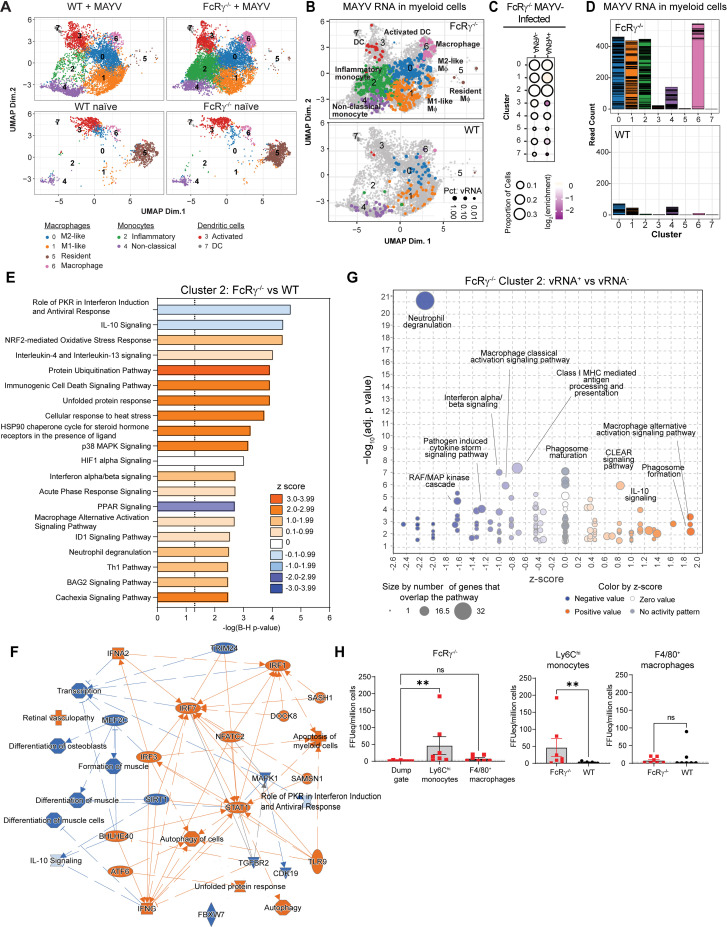
Distinct transcriptomic changes in FcRγ−/− monocytes during resolution of MAYV infection. (A) Subcluster analysis was performed on the myeloid cell subset, which is indicated by the dashed line in (Fig 4B). (B) Cells expressing MAYV RNA in the myeloid subcluster are colored based on the subcluster analysis and the size denotes the percentage of viral RNA in the cell. (C) The proportion of cells from the FcRγ^−/−^ myeloid subclusters, separated by the presence of MAYV RNA, showing the log_2_(enrichment) for each cluster between viral RNA positive and negative cells. (D) Total MAYV RNA read count per cell in the myeloid subcluster analysis. Each horizontal bar represents a single cell. (E-G) Ingenuity pathway analysis on differentially expressed genes in FcRγ^−/−^ monocytes (Cluster 2) or FcRγ^−/−^ vRNA+ monocytes using an enrichment cutoff of Log2FC ≤ -0.58 or ≥ 0.58 and an adjusted P value < 0.05. (E) Enriched canonical pathways and (F) graphical summary of predicted pathway activity in FcRγ^−/−^ monocytes compared to WT mice. (E and G) Orange bars or dots indicate a positive z-score, blue bars or dots indicate a negative z-score, white bars or dots represent a z-score of 0, and a z-score could not be defined in gray dots. (F) Orange nodes/lines indicate predicted activation and blue nodes/lines indicate predicted inhibition. Relationships between nodes are distinguished by the lines. A solid line leads to activation/inhibition, a thin dashed line is an inferred relationship, and a grey line represents a direct interaction. (H) Four-week-old WT or FcRγ^−/−^ C57BL/6N mice were infected with MAYV, and the ipsilateral foot and surround skin were processed as described above at 10 dpi (n = 8 per group; 2 independent experiments). Single cell suspensions were stained and sorted for Ly6C^hi^ monocytes (Live, CD45^+^, CD11b^+^, Ly6c^hi^) and F4/80^+^ macrophages (Live, CD45^+^, CD11b^+^, Ly6c^mid-low^, F4/80^+^). The remaining CD45^+^ cells that did not fall within those parameters were collected as a Dump gate (Live, CD45^+^, CD11b^-^, NK1.1^+/−^). MAYV RNA was isolated from the sorted cells and quantified by RT-qPCR with virus specific primers and probe. The dotted line indicates the limit of detection for the assay. Statistical significance was determined by a Kruskal-Wallis test with a Dunn’s post-test (left graph) or Mann-Whitney test (right two graphs) (**, ****P**** < 0.01; ns = not significant).

Focusing on the cellular populations that were shown to be both altered (Fig 3) as well as contained MAYV RNA, we reanalyzed the clusters outlined in the black dashed line (Clusters 0, 1, 2, 3, 4, 8, 16) (Fig 4B) and identified the myeloid cell subsets using key gene markers (S6A–S6C Fig). A clear distinction between MAYV-infected and naïve mice was observed, with the loss of tissue-resident macrophages (Cluster 5) and an increase in the clusters identified as monocytes, activated macrophages, and dendritic cells (Figs 5A and S6D). There were no substantial differences in the clusters identified in the naïve groups, suggesting similar myeloid populations are present at baseline (S6D Fig). Compared to infected WT mice, infected FcRγ^−/−^ mice had a dramatic increase in the number of inflammatory monocytes (Cluster 2) (Fig 5A), which is consistent with our flow cytometry results (Fig 3A and 3B).

Evaluation of MAYV RNA distribution revealed an increased presence and enrichment of viral RNA in the FcRγ^−/−^ mice across multiple clusters (Figs 5B–5D, and S6E). M1-like macrophages (Cluster 1) from FcRγ^−/−^ mice were enriched in the viral RNA positive cells compared to the negative cells. This was distinct from the WT mice where non-classical monocytes (Cluster 4) were enriched in the viral RNA positive cells (S6E Fig). For both groups, there was a negative enrichment for Cluster 3, 6, 7 in the viral RNA positive cells suggesting these cells are actively preventing infection (S6E Fig). In WT mice, inflammatory monocytes (Cluster 2) were also negatively enriched in viral RNA containing cells; however, this may be due to the reduced number of cells present in the cluster (Figs 5B and S6E). On a per cell basis, the macrophages (Clusters 0, 1, and 6) and monocytes (Clusters 2 and 4) from FcRγ^−/−^ mice had the most MAYV RNA reads, albeit the majority of the MAYV reads in Cluster 6 are derived from one cell (Fig 5D). Although the MAYV RNA read count was lower in the WT mice, most of the reads grouped within Clusters 0, 1, and 4 suggesting a differential enrichment of MAYV RNA in inflammatory monocytes (Cluster 2) and macrophages (Cluster 6) in the FcRγ^−/−^ mice (Fig 5D). Overall, these results show enriched MAYV RNA in myeloid cells that lack activating FcγRs.

### Distinct transcriptomic changes in FcRγ ^−/−^ monocytes during resolution of MAYV infection

Previous studies have implicated monocytes and macrophages as potential targets of alphavirus infection which may impact the cellular response [65–67]. To interrogate the differences in the monocyte (Cluster 2) response, which may have contributed to the increased viral RNA, we performed IPA on DEGs enriched in the FcRγ^−/−^ mice compared to WT mice. We tried to perform IPA on Cluster 6, however only 10 genes met the cutoff for enrichment (Log2FC ≤ -0.58 or ≥ 0.58, adjusted P value < 0.05) which resulted in no predicted Z scores for any of the enriched pathways. This suggests that this macrophage population is similar between the groups. For Cluster 2, up-regulated pathways (z score > 0) included broad categories of cellular stress response, protein ubiquitination pathways, type I interferon signaling, cytokine and chemokine signaling, and cellular differentiation (Fig 5E and 5F). The down-regulated pathways (z score < 0) involved protein kinase R (PKR) induction, IL-10 signaling, and peroxisome proliferator activating receptor (PPAR) signaling (Fig 5E and 5F). When enriched pathways were compared between the myeloid subclusters from the FcRγ^−/−^ mice, similar gene ontology pathways were identified, suggesting that the transcriptional landscape within these cell populations is similar (S7A Fig).

Within the FcRγ^−/−^ mice, MAYV RNA^+^ monocytes showed distinct DEGs as well as pathway enrichment compared to MAYV RNA^-^ monocytes (Figs 5G, S7B and S7C). vRNA^+^ monocytes were enriched for proinflammatory *Cd40* and *Ccl12* transcripts and negatively enriched for *Ccl5*, *Ccl7* (S7B Fig). IPA showed positive Z scores for pathways associated with non-classical macrophage activation/polarization and phagosome formation while down-regulated pathways included MHC class I processing and presentation, type I interferon signaling, classical macrophage activation, cytokine signaling, and degranulation (Fig 5G). These results suggest that the monocytes containing MAYV RNA are transcriptionally distinct and are undergoing an alternative activation pathway.

To validate the increased MAYV RNA observed in specific FcRγ^−/−^ cellular clusters, we sorted Ly6C^hi^ monocytes, F4/80^+^ macrophages, and any remaining CD45^+^ cells (Dump gate) from the ipsilateral ankle of MAYV infected animals at 10 dpi and quantified MAYV E2 RNA by RT-qPCR. Within the FcRγ^−/−^ sorted cells, MAYV RNA was enriched in Ly6C^hi^ monocytes (Fig 5H *left*). Between the FcRγ^−/−^ and WT groups, Ly6C^hi^ monocytes had significantly more MAYV RNA with no difference in the level of MAYV RNA in F4/80^+^ macrophages (Fig 5H *middle and right*). To determine if the FcRγ^−/−^ monocytes or macrophages were contributing to the increased viral burden in the ipsilateral ankle (Fig 1C), we sorted Ly6C^hi^ monocytes and F4/80^+^ macrophages from the ipsilateral ankle of MAYV infected animals at 10 dpi and cultured serial dilutions of the cells on C6/36 cells to amplify any released infectious virions. The presence of MAYV was determined by FFA on the supernatant 72 hours post co-culture. No infectious virus was recovered from monocytes or macrophages isolated from either the WT or FcRγ^−/−^ mice. These results confirm the enrichment of MAYV RNA in Ly6C^hi^ monocytes and suggest the vRNA^+^ monocytes may not be the source of enhanced infectious MAYV in the joint-associated tissue but instead are promoting a pro-viral environment.

### Monocytes lacking activating FcγRs are sufficient to drive prolonged MAYV infection

**Fig 6:**
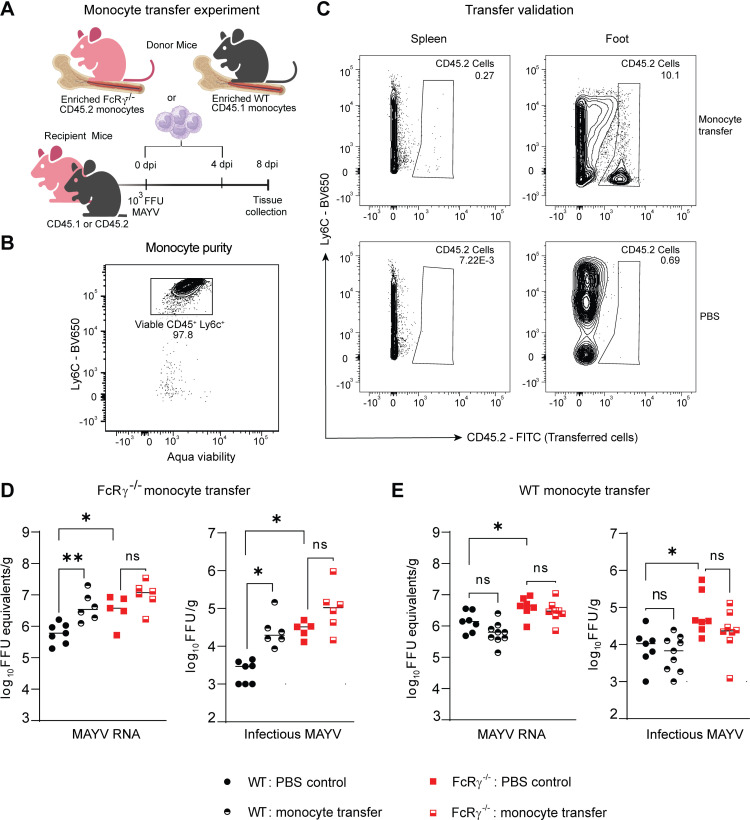
Monocytes lacking activating FcγRs enhance viral infection. (A) Schematic of monocyte transfer experiment. Monocytes were enriched through negative selection from the bone marrow of either FcRγ^−/−^ CD45.2 or WT CD45.1 mice. Recipient WT and FcRγ^−/−^ mice were injected intravenous with a PBS control or either WT or FcRγ^−/−^ monocytes at 0 dpi (5 x 10^5^ monocytes) and 4 dpi (1 x 10^6^ monocytes). This figure was created in Biorender.com using a publication license. (B) Representative flow plots show Ly6C^hi^ expression of the monocytes enriched from bone marrow prior to transfer into recipient mice. (C) Representative flow plots show CD45.2 donor cells identified in single cell suspensions from either the spleen or ipsilateral ankle of CD45.1 recipient mice. (D-E) Quantification at 8 dpi of MAYV RNA and infectious virus from the ipsilateral ankle of mice receiving (D) FcRγ^−/−^ or (E) WT monocytes compared to PBS control. Bars indicate median values (n = 5 to 9; 2 to 3 independent experiments; one-way ANOVA with a Tukey’s post-test (MAYV RNA), Kruskal-Wallis test with a Dunn’s post-test (Infectious MAYV); *, ****P**** < 0.05; **, ****P**** < 0.01).

Earlier results showed that FcRγ^−/−^ mice had increased Ly6C^hi^ monocytes in the ipsilateral foot through 28 dpi (Fig 3) and contained more MAYV RNA at 10 dpi (Fig 5B and 5D). Monocytes have previously been implicated in both tissue damage and/or disease resolution following alphavirus infection, shown to be productive targets of MAYV infection, and promote infection of non-hematopoietic cells within the tissue during CHIKV infection [37,66,68–70]. To determine if FcRγ^−/−^ monocytes were sufficient to increase MAYV in the joint-associated tissue, FcRγ^−/−^ monocytes (CD45.2) were enriched from the bone marrow of donor mice, transferred into MAYV-infected CD45.1 (WT) or CD45.2 (FcRγ^−/−^) recipient mice at 0 and 4 dpi, and MAYV was quantified from the ipsilateral ankle of recipient mice at 8 dpi (Fig 6A). The purity of the isolated monocytes was confirmed prior to adoptive transfer (Fig 6B) and the presence of transferred cells in WT animals was confirmed in the spleen and ipsilateral ankle at 8 dpi by flow cytometry (representative plot, Fig 6C). When the transferred monocytes were identified 8 dpi in the ipsilateral ankle, the cells showed a noticeable reduction in Ly6C expression likely indicating the cells are differentiating into a monocyte-derived DC or macrophage. Consistent with previous data, lack of activating FcγRs led to increased MAYV infectious virus and RNA in PBS control mice (Fig 6D and 6E). Transfer of the FcRγ^−/−^ monocytes significantly increased the level of MAYV RNA and infectious virus in the WT mice compared to the PBS control treated WT mice (Fig 6D). This trend was also observed when the FcRγ^−/−^ monocytes were transferred back into the FcRγ^−/−^ mice (Fig 6D). In contrast, transfer of WT monocytes (CD45.1) into MAYV-infected WT or FcRγ^−/−^ CD45.2 recipient mice showed minimal change in infectious virus or viral RNA between monocyte transfer and PBS control for each genotype (Fig 6E). Taken together, these data show that monocytes lacking activating FcγRs are sufficient to prolong MAYV burden in joint-associated tissue at 8 dpi, most notably in mice that also have functional monocytes, indicating a dominant pro-viral role for these cells.

## Discussion

The generation of antiviral antibodies during alphavirus infection is critical for clearance of infectious virus; however, the importance of Fc effector functions to mediate this reduction was unknown. Here, we examined the role of activating FcγRs during MAYV infection and determined that activating FcγRs are necessary for optimal resolution of clinical disease and reduction of MAYV RNA through 28 dpi. This was most notable for joint-associated tissues, which was not observed previously for CHIKV [27]. Analysis of antibody responses showed that despite equivalent neutralization and quantity at a chronic time point, a significant increase in MAYV RNA remained in the tissue. Despite early differences in neutralizing antibodies, we showed that neutralizing antibodies were not sufficient to enhance viral control in FcRγ^−/−^ mice. Only mice lacking antibodies or specifically Fc-FcγR engagement increased viral burden in the joint-associated tissue showcasing the importance for MAYV binding antibodies during resolution. Prolonged monocyte infiltration as well as distinct genetic signatures in these cells were observed in FcRγ^−/−^ mice, and, when transferred into WT mice, the FcRγ^−/−^ monocytes were sufficient to increase levels of both infectious virus and viral RNA at a time point associated with disease resolution. Taken together, these data demonstrate the necessity of activating FcγR signaling, specifically on monocytes, for viral control following MAYV infection.

Fc effector functions have been shown to be important for protection from a variety of viruses [22,71,72]. In mice, NK cells, neutrophils, DCs, and monocytes express combinations of activating FcγRs (FcγRI, FcγRIII, and FcγRIV) that facilitate opsonization, antibody-dependent cellular cytotoxicity (ADCC), antibody-dependent cellular phagocytosis (ADCP), and/or enhance T cell activation through DC maturation via interaction with the Fc region of IgG [73]. Previously, engagement of FcγRs on monocytes with anti-alphavirus mAbs was shown to be critical for clearance of CHIKV and MAYV RNA [18,22,29,74]. Furthermore, non-neutralizing anti-MAYV mAbs increased phagocytosis of immune complexes into FcγR-bearing myeloid cells resulting in an abortive infection and clearance of the virus [30]. Despite lacking activating FcγRs in our model, the inhibitory FcγR (FcγRIIb) is still present in the FcRγ^−/−^ mice since this receptor does not signal through the Fc receptor common gamma chain. Furthermore, the inhibitory FcγR can also be expressed on myeloid cells [33]. Previous work has shown an enhanced inflammatory response in the absence of FcγRIIb signaling [75]; however, the impact of only expressing FcγRIIb during infection is less understood.

In our model, mice lacking the activating FcγRs showed prolonged disease and MAYV burden through chronic time points even in the presence of neutralizing antibodies. A detailed characterization of the humoral response showed increased IgG2c and reduced IgG2b in FcRγ^−/−^ mice at 8 dpi, but the levels were equivalent by 28 dpi. A previous report showed that IgG2c has a higher affinity for FcγRI and FcγRIV compared to IgG2b and IgG2b has increased binding to FcγRIII compared to IgG2c [76]. While IgG2c was shown to be superior at clearing antigen from circulation than IgG2b, the mechanism was receptor dependent which is not applicable in our FcRγ^−/−^ mice [76]. Both IgG2b and IgG2a/c have a similar low binding affinity for the inhibitory FcγRIIb indicating that mice lacking activating FcγRs do not preferentially produce antibodies that target FcγRIIb (*e.g.,* IgG1) [77]. Notwithstanding, interaction of the antiviral antibodies with FcγRIIb, in the absence of signaling through the activating FcγRs, may have resulted in differential cellular responses. Interestingly, Jh mice, which fail to produce antibodies, had equivalent viral burden as the FcRγ^−/−^ mice in joint-associated tissues highlighting the host reliance on Fc-FcγR interactions for tissue-specific MAYV clearance rather than neutralizing antibodies. More broadly, studies have shown Fc-FcγR interaction on monocytes has also been implicated for enhanced mAb-mediated clearance during SARS-CoV-2 infection [78,79], suggesting a more broad importance of these interactions and a need to characterize them.

Despite the prolonged swelling and increased viral burden in FcRγ^−/−^ mice infected with MAYV, infectious virus is eventually cleared and swelling resolves. In the absence of activating FcγR engagement, the increased infiltration of inflammatory immune cells, such as monocytes and neutrophils, may compensate for the lack of Fc-mediated viral clearance at the cost of increased clinical disease. In line with this, a positive correlation has been observed between monocyte chemoattractant protein-1 (MCP-1; CCL2) levels and severe disease following CHIKV infection in humans [80]. Additionally, inhibition of MCP-1 reduced CHIKV disease in mouse models [81]. However, mice lacking the CCL2 receptor (CCR2^−/−^) or antibody-mediated depletion of CCR2^+^ inflammatory monocytes resulted in increased disease during MAYV, RRV, or CHIKV infections, likely due to a compensatory influx of neutrophils and the potential for monocytes to still traffic into tissues through CCR7 [69,82–84]. Additionally, CD8^+^ T cells, which have previously been shown to control infectious virus in muscle tissue during RRV infection, are more numerous through 28 dpi and appear more transcriptionally active in the FcRγ^−/−^ mice at 10 dpi [64]. This suggests that the increased presence of CD8^+^ T cells cannot compensate for the absence of activating FcγRs, as MAYV RNA is significantly increased in various tissues of FcRγ^−/−^ mice at 28 dpi despite clearance of infectious virus and disease resolution.

Of particular interest was the inversion in the monocyte to macrophage ratio. By 10 dpi, WT mice had reduced numbers of monocytes with increased number of macrophages compared to FcRγ^−/−^ mice. Macrophages, which can be monocyte-derived, respond rapidly to stimuli from the environment and enact a diverse range of effector functions. Monocytes and macrophages have been shown to provide protective and pathogenic roles during alphavirus infection. Monocytes produce type I interferons following alphavirus recognition, which has been shown as necessary for controlling viral replication [74,82,85]. Conversely, depletion of phagocytes through clodronate-loaded liposomes prior to CHIKV infection reduced clinical disease [37]. Monocytes and macrophages have also been identified as targets of alphavirus infection [65,70,86,87] and specifically for MAYV [66,88]. A recent study showed reduced MAYV burden in CCR2^−/−^ mice [84]. While it was hypothesized that the reduced viral load in the CCR2^−/−^ mice was related to an increase in neutrophil recruitment, it is possible that the recruited monocytes are an important source of infectious virus. Additionally, infiltrating monocytes are productive targets of CHIKV infection and interestingly can increase infection in non-immune cells at the site of infection [70]. In the single-cell RNA sequencing analysis, the CD45^+^ cells contained more MAYV RNA compared to the CD45^-^ cells. This finding was surprising as CHIKV is known to replicate in CD45^-^ cells such as fibroblasts, epithelial cells, myoblasts [89]. While the cellular tropism for MAYV is not as defined, it is anticipated that similar cell types would be targeted. Alternatively, some CD45^-^ cells may have been lost due to the tissue digestion for single-cell RNA sequencing. Notwithstanding, monocytes and macrophages can also phagocytose virally infected cells and virions, so the presence of viral RNA does not necessarily equate to a productive infection. However, phagocytosis will most likely be reduced in the absence of activating FcγRs. Whether the FcRγ^−/−^ monocytes are infected or acquiring vRNA through phagocytosis remains unclear and future studies are needed.

Pathway analysis of the inflammatory monocyte subcluster showed enrichment of pathways in the FcRγ^−/−^ mice utilized by alphaviruses for efficient viral replication, including proteasome-ubiquitination, oxidation, and heat stress response pathways [90–92]. Additionally, pathways associated with type-I IFN and antiviral responses were enriched. Interestingly, pathway analysis comparing vRNA^+^ cells to vRNA^-^ cells within the FcRγ^−/−^ inflammatory monocyte subcluster did not enrich for cellular stress, but rather were positively enriched for pathways related to alternative activation and phagosome formation. This suggests a role of phagocytosis mediated uptake of MAYV through receptors other than FcγRs, rather than infection, which may facilitate cellular differentiation of the monocytes to a M2-like macrophage and reduced viral clearance [93]. Additionally, there was increased expression of *Ccl12* in the vRNA^+^ cells. CCL12 binds to CCR2 and has been implicated in promoting bone resorption [94] which has been observed following alphavirus infection [95] and may contribute to the increased disease observed in the FcRγ^−/−^ mice.

Adoptive transfer of FcRγ^−/−^ monocytes into WT or FcRγ^−/−^ mice increased MAYV burden while transfer of WT monocytes did not reduce MAYV load in FcRγ^−/−^ mice. Ex vivo culture of sorted monocytes and macrophages from the ipsilateral feet of WT and FcRγ^−/−^ mice failed to produce infectious virus suggesting that these cells are not a dominant source of infectious virus. Although, this comes with the experimental caveat that potentially too few monocytes/macrophages were plated to observe virus amplification. Nonetheless, the adoptive transfer study suggests that there are intrinsic differences in the FcRγ^−/−^ monocytes that result in a dominant, pro-viral response during MAYV infection that may augment local infection rather than just failing to clear infected cells and virions via Fc-FcγR interactions.

Taken together, the pathway analysis results and the increased infectious MAYV following transfer of FcRγ^−/−^ monocytes suggest that lack of activating FcγR signaling alters the response of monocytes and macrophage to MAYV infection resulting in an enhanced pro-viral environment. Future studies are needed to interrogate specific signaling pathways that mediate this prolonged infection, which may provide insight into factors that promote responsiveness of myeloid cells to infections and ultimately how Fc-FcγR interactions modify cellular responses. The results from this study indicate that the pathogenic or protective functions of monocytes may result from a more nuanced relationship between antiviral antibodies and their interaction with the activating FcγRs.

## Materials and methods

### Ethics statement

All animal experiments and procedures were carried out in accordance with the recommendations in the Guide for the Care and Use of Laboratory Animals of the National Institutes of Health and approved by the National Institute of Allergy and Infectious Diseases (NIAID) Animal Care and Use Committee (ACUC) under the protocol LVD 6E. Four-week-old male and female C57BL/6NTac (WT), B6.129P2-*Fcer1g*^*tm1Rav*^N12 (FcRγ^−/−^), C57BL/6NTac-*Igh-J*^*em1Tac*^ (Jh), and B6.SJL-*Ptprc*^*a*^/BoyAiTac (all strains from Taconic Biosciences) were used for our studies. Footpad inoculations were performed under anesthesia that was induced and maintained with isoflurane. Retro-orbital intravenous injections were performed under anesthesia with 2, 2, 2-tribromoethanol (Avertin). Mice were inoculated subcutaneously in the rear footpad with 10^3^ FFU of MAYV, RRV, or CHIKV diluted in Hanks’ Balanced Salt Solution (HBSS, Gibco) supplemented with 1% HI-FBS. Foot swelling was measured (width x height) prior to infection and on indicated time points using digital calipers. Mice were sacrificed, perfused with PBS, and tissues collected at 3, 8, 10, or 28 dpi.

### Cells and viruses

Vero cells (African Green Monkey Kidney, female; ATCC) were cultured in Dulbecco’s Modified Eagle Medium (DMEM) (Gibco) supplemented with 5% heat-inactivated fetal bovine serum (HI-FBS; Omega) at 37°C with 5% CO_2_. Mayaro virus isolate BeH407 was received from the World Reference Center for Emerging Viruses and Arboviruses (WRCEVA) and passaged twice on Vero cells. Ross River virus strain T48 was produced from an infectious cDNA clone, as described previously, and passaged once on BHK cells [96]. Chikungunya virus strain AF15561 was produced from an infectious cDNA clone, as previously described, and passaged once on BHK cells [97].

### Focus forming assay (FFA)

Tissues were weighed then homogenized in DMEM supplemented with 2% HI-FBS, 10mM HEPES (Gibco) and penicillin and streptomycin (Gibco) using silica beads. Homogenates were clarified (12,000 × rpm for 5 min). Vero cells, plated in 96-well flat bottom plate one day prior, were infected with serial dilutions of clarified tissue homogenates for 2 hours at 37°C. The inoculum was removed, then the cells were overlayed with a 1% methylcellulose (Sigma-Aldrich) in Minimum Essential Medium (MEM, Sigma-Aldrich) supplemented with penicillin and streptomycin, 10 mM HEPES, and 2% HI-FBS. Cells were fixed 18 h later with 4% paraformaldehyde (PFA; Electron Microscopy Sciences) in PBS. Cells were washed with PBS, permeabilized with perm wash (PBS supplemented with 0.1% saponin and 0.1% BSA) and stained using CHK-48 [19]. Following a wash with ELISA wash buffer (PBS with 0.05% Tween-20), cells were incubated with peroxidase-conjugated goat anti-mouse IgG (H + L) antibody (SeraCare) for 1-2 h. Cells were washed with ELISA wash buffer and foci were developed using TrueBlue substrate (KPL) and counted using a Biospot plate reader (Cellular Technology, Inc.). Viral titers were normalized to tissue weight.

### Quantification of viral RNA

RNA was isolated using the KingFisher Duo Prime System with the MagMAX Viral RNA Isolation Kit (Applied Biosystems) following the manufacturer’s instructions. Viral RNA was quantified by qRT-PCR using the TaqMan Fast Virus 1-Step MasterMix with RRV nsP3 specific primers (Forward: 5’ - GTG TTC TCC GGA GGT AAA GAT AG -3’, Reverse: 5’ - TCG CGG CAA TAG ATG ACT AC - 3’) and probe (5’ - 6FAM/ACC TGT TTA/ZEN/CCG CAA TGG ACA CCA/ 3IABkFQ/ - 3’), CHIKV E1 specific primers (Forward: 5’ – TCG ACG CGC CAT CTT TAA – 3’, Reverse: 5’ – ATC GAA TGC ACC GCA CAC T – 3’) and probe (5’ – 6FAM/GCC GAG AGC/ZEN/CCG TTT TTA AAA TCA C/3IABkFQ – 3’), or MAYV E2 specific primers and probe (Forward: 5’ – GTG GTC GCA CAG TGA ATC TTT C- 3’, Reverse: 5’ – CAA ATG TCC ACC AGG CGA AG – 3’, Probe: 5’ - 6FAM/ATG GTG GTA/ZEN/GGC TAT CCC ACA GGT C/3IABkFQ – 3’)and compared to RNA isolated from viral stocks as a standard curve to determine FFU equivalents. Viral RNA was normalized to tissue weight.

### Focus reduction neutralization test (FRNT)

Serum from infected animals was serially diluted in DMEM supplemented with 2% HI-FBS, penicillin and streptomycin, and 10 mM HEPES and incubated with 10^2^ FFU MAYV for 1 h at 37°C in duplicate wells. Serum-virus mixtures were added to Vero cells for 90 min at 37°C followed by an overlay with a 1% methylcellulose in Minimum Essential Medium (MEM, Invitrogen) supplemented with penicillin and streptomycin, 10 mM HEPES, and 2% HI-FBS. Cells were fixed 18 h later after the addition of 4% PFA in PBS. Infected cells were incubated with CHK-48 (0.5 µg/ml). After washing and incubation with peroxidase-conjugated goat anti-mouse IgG (SeraCare), foci of infection were developed using TrueBlue substrate (KPL) and counted using a Biospot plate reader (Cellular Technology, Inc.). Wells containing serum dilutions were compared to wells inoculated in the absence of serum. The half maximal inhibitory serum dilution (Neut_50_ value) was calculated using non-linear regression analysis constraining the bottom to 0 and top to 100.

### Quantification of anti-MAYV antibodies from serum

For direct ELISAs, recombinant MAYV E2 protein (Native Antigen) was absorbed overnight at 4°C on Maxisorp immunocapture ELISA plates (Thermo Scientific) in a sodium bicarbonate buffer pH 9.3. Wells were washed with ELISA wash buffer and blocked with blocking buffer [PBS + 2% BSA (Sigma)] for 2 h at 37°C. Mouse serum was heat inactivated at 56°C for 1 h, serially diluted in blocking buffer, and then added to wells for 2 h at room temperature (RT). Plates were washed with ELISA wash buffer then incubated for 1 h at RT with an HRP conjugated goat anti-mouse for either total IgG or specific IgG subclasses (Southern Biotech). Plates were washed with ELISA wash buffer and developed using 1-Step Ultra TMB-ELISA substrate solution (Thermo Fisher). The reaction was stopped with 1 M H_2_SO_4_ and absorbance was measured at 450 nm. The EC_50_ of each sample was calculated using non-linear regression analysis after constraining the bottom to 0 and the top to 100 in GraphPad Prism.

For MAYV capture ELISAs, RRV-12 (Leinco Technologies), a human anti-RRV mAb that cross reacts with MAYV [21], was coated on immunocapture ELISA plates, blocked, and washed as above. MAYV was captured for 2 hours at RT and washed before incubating with serial diluted mouse serum as above. HRP conjugated goat anti-mouse IgG (southern biotech) or IgM (Jackson ImmunoResearch) was used as the secondary antibody.

### Antibody binding avidity assay

To measure antibody avidity, a chaotropic ELISA was performed. ELISA detecting total IgG against directly coated recombinant E2 was performed as above except after incubation of serum antibody samples and washing, the wells were incubated for 20 minutes at RT with either 5M urea, 3M urea, or PBS. Plates were then washed three times with ELISA wash buffer and reblocked with blocking buffer for 1 h at 37°C before continuing the ELISA as above. Relative avidity index (RAI) was calculated for each sample by dividing the OD at 450 nm in urea-treated wells by that in untreated wells (PBS) for each individual sample. Samples with a RAI of >60% were considered to have high avidity, 40% to 60% medium avidity, and <40% was low avidity [98].

### ADCC reporter assay

Vero cells were seeded at a density of 2×10^4^ cells per well in white 96 well flat-bottom plates. The following day cells were infected with MAYV BeH407 at an MOI of 2 in infection media for 1 h, washed with PBS, then fresh infection media was added. Four hours later, the cells were rinsed and assay buffer (RPMI 1640 supplemented with 4% low IgG FBS (Gibco)) was added to each well followed by diluted mouse serum (final dilution of 1:40 in assay buffer performed in duplicate). After incubation for 1.5 h at 37°C with 5% CO_2_, Jurkat effector cells expressing murine FcγRIV (Promega) were resuspended in assay buffer were added to each well at an effector cell: target cell ratio of 5:1. The cells were incubated for an additional 16 h at 37°C with 5% CO_2_ before luciferase activity was evaluated using the Bio-Glo Luciferase assay (Promega) and following the manufacturer’s instructions. Briefly, Bio-Glo Luciferase Assay Reagent (Promega) and assay plate were brought to RT. The Bio-Glo Luciferase Assay Reagent was added to each well and incubated at RT. Luminescence in relative light units (RLU) was quantified using a Synergy H1 plate reader (Biotex). Background RLU was subtracted from each sample. Fold induction was obtained by dividing the RLU of the wells of interest by the mean of control wells containing infected target cells and Jurkat effector cells with no serum.

### Antibody depletion of B cells

Mice were administered two doses of 500 μg mouse anti-mouse CD20 (BioXcell, clone MB20-11; cat# BE0356) or an isotype control (BioXcell, IgG2c) at 0 and 4 days post-infection by intraperitoneal injection. At 8 dpi, the ipsilateral and contralateral ankles were collected, homogenized, and viral burden was assessed by FFA as described above. Cells from peripheral blood were isolated and washed with FACS buffer (PBS with 1% BSA) and single cell suspensions were blocked for FcγR binding (BioLegend clone 93; 1:50) and then stained for 1 h at 4°C with the following antibodies to validate B cell depletion: CD45 BUV395 (BD Biosciences clone 30-F11; 1:200), CD19 BUV737 (BD clone 1D3; 1:300), and B220/CD45R BV421 (Biolegend clone RA3-6B2; 1:200). Cells were washed 3 times in FACS buffer then fixed in 4% PFA in PBS for 10 minutes at 4°C. Cells were then washed and resuspended in FACS buffer. Viability was determined through exclusion of a fixable viability dye (aqua). Samples were processed on a BD LSRFortessa (BD Biosciences) and analyzed using FlowJo (FlowJo, LLC).

### Serum IgG binding to live MAYV-infected cell surface

Vero cells were seeded at 2.5 x 10^4^ cells/well in 96 well plates and infected with MOI 5 MAYV for 18 h at 37°C with 5% CO_2._ Cells were then trypsinized until detached and resuspended in FACS buffer (PBS with 1% BSA). Cells were then incubated with serum diluted in FACS buffer (1:200) for 1 h at 4°C. Cells were washed 3 times in FACS buffer and then stained with an AF647-conjugated anti-mouse IgG secondary antibody (eBiosciences; 1:2000). Cells were washed 3 times in FACS buffer then fixed in 4% PFA in PBS for 10 minutes at 4°C. Cells were then washed and run on a BD LSRFortessa and analyzed using FlowJo.

### Flow Cytometry

Mice were infected with 10^3^ FFU of MAYV and at 3, 8, or 10 dpi mice were sacrificed and perfused with PBS. The ipsilateral feet were disarticulated, and the skin was everted. The skin was minced then the tissue (feet and skin combined) was digested in RPMI 1640 supplemented with collagenase (2.5mg/mL; Sigma), Liberase (100ug/mL; Roche), HEPES (15mM), and DNase I (10ug/mL; Sigma) for 2 h at 37°C with agitation, strained through a 70-μm filter, and resuspended in RPMI 1640 supplemented with 10% HI-FBS. Cells were then washed with FACS buffer, single cell suspensions were blocked for FcγR binding (BioLegend clone 93; 1:50), then stained with the following anti-mouse antibodies for 1 h at 4°C: CD45 BUV395 (BD Biosciences clone 30-F11; 1:200), CD3 AF488 (BioLegend clone KT3.1.1; 1:400), CD4 AF647 (eBiosciences clone RM4-5; 1:400), CD8b AF700 (eBiosciences clone YTS156.7.7; 1:400), NK1.1 PE-Cy7 (BioLegend clone PK136; 1:200), CD11b PerCP-Cy5.5 (BioLegend clone M1/70; 1:200), CD19 BUV737 (BD clone 1D3; 1:300), Ly6C BV650 (BioLegend clone HK1.4; 1:400), Ly6G APC-Cy7 (BioLegend clone 1A8; 1:300), CD11c PE-Cy5 (BioLegend clone N418; 1:400), I-A/I-E (MHC class II) BV711 (BioLegend clone M5/114.15.2; 1:400), and F4/80 BV421 (BD Biosciences clone T45-2342; 1:400). Cells were washed 3 times in FACS buffer then fixed with 4% PFA in PBS for 10 minutes at 4°C. Cells were then washed and resuspended in FACS buffer. Viability was determined through exclusion of a fixable viability dye (aqua). Samples were processed on a BD LSRFortessa and analyzed using FlowJo.

For cell sorting experiments validating MAYV RNA in specific immune cell populations, cells were stained as above with CD45 BUV395 (BD Biosciences clone 30-F11; 1:200), CD11b PerCP-Cy5.5 (BioLegend clone M1/70; 1:200), Ly6C BV650 (BioLegend clone HK1.4; 1:400), Ly6G APC-Cy7 (BioLegend clone 1A8; 1:300), NK1.1 PE-Cy7 (BioLegend clone PK136; 1:200), and a fixable viability dye (aqua). Cell populations were sorted on a BD FACSAria as “Ly6C^hi^ monocytes” (Live, CD45^+^, CD11b^+^, Ly6C^hi^), “F4/80^+^ macrophages” (Live, CD45^+^, CD11b^+^, Ly6C^mid-low^, F4/80^+^), or “Dump gate” (Live, CD45^+^, CD11b^-^, NK1.1^+/−^). Sorted cells were pelleted and RNA isolated with a Qiagen RNeasy RNA isolation kit following the manufacturer’s protocol. Virus specific RNA was quantified using MAYV E2 primers, as described above, and viral FFU equivalents was calculated using a standard curve then normalized to number of cells collected in each population.

### Adoptive transfer of monocytes

The bone marrow from tibias and femurs of donor C57BL/6N CD45.1 or FcRγ^−/−^ CD45.2 mice was aspirated and collected in RPMI 1640 (Invitrogen) at 4°C. Monocytes from bone marrow were enriched by negative selection (Monocyte Isolation Kit BM, Miltenyi Biotec) following the manufacturer’s instructions and resuspended in sterile PBS (Gibco). Monocyte purity was confirmed by staining with CD45 BUV395 (BD Biosciences clone 30-F11; 1:200), CD11b PerCP-Cy5.5 (BioLegend clone M1/70; 1:200), Ly6C BV650 (BioLegend clone HK1.4; 1:400) and CD11b PerCP-Cy5.5 (BioLegend clone M1/70; 1:200) as described above. Negatively enriched monocytes were intravenously infused into C57BL/6N WT or FcRγ^−/−^ CD45.2 recipient mice at 0 dpi (5 x 10^6^ cells) and 4 dpi (1 x 10^6^ cells). At 8 dpi, the ipsilateral ankles were collected, homogenized, and viral burden was assessed by FFA and qRT-PCR as described above. The spleen and ipsilateral feet were collected to confirm monocyte transfer. The feet were digested, as described above. The spleen was passed through a 70 µm filter then rinsed with RPMI 1640 supplemented with 10% HI-FBS. Cells were then washed with FACS buffer, single cell suspensions were blocked for FcγR binding (BioLegend clone 93; 1:50), then stained with the following anti-mouse antibodies for 1 h at 4°C: CD45.1 BUV395 (BD Biosciences clone A20; 1:200), CD45.2 FITC (BD Biosciences clone 104; 1:200), CD11b PerCP-Cy5.5 (BioLegend clone M1/70; 1:200), and Ly6C BV650 (BioLegend clone HK1.4; 1:400). Cells were washed 3 times in FACS buffer then fixed with 4% PFA in PBS for 10 minutes at 4°C. Cells were then washed and resuspended in FACS buffer. Viability was determined through exclusion of a fixable viability dye (aqua). Samples were processed on a BD LSRFortessa and analyzed using FlowJo.

### Single-cell RNAseq preparation and analysis

Mice were infected with 10^3^ FFU of MAYV and, at 10 dpi, mice were sacrificed, perfused with PBS, and the ipsilateral feet were dissociated into a single cell suspension, as described above. Cells were stained with anti-CD45 BUV395 (BD Biosciences clone 30-F11; 1:200) and a viability dye. Viable, unfixed, CD45^+^ or CD45^-^ cells were sorted on a BD FACSAria into RPMI supplemented with 10% FBS. Sorted CD45^+^ immune cells were centrifuged and resuspended in 1X PBS with BSA 0.04% to achieve 1000 cell/µL concentration. For the preparation of the cDNA and sequencing library generation, we followed manufacturer instructions from the Chromium Next GEM Single Cell 5’ Reagent Kit v2 (Dual index) User Guide with one modification: primers targeting the non-structural and subgenomic RNA were spiked-in during the cDNA synthesis to capture viral RNA (Step 1.1). The virus-specific primer concentration added to each RT reaction was ~15 pmoles [99]. All the other reagents were added according to the protocol, except the nuclease-free water, which was reduced to accommodate the primer spike-in volume. The Illumina library quality, yield, and size distribution was assessed by TapeStation D1000 high sensitivity assay, and by Qubit High Sensitivity dsDNA kit. The molar concentrations of the libraries were determined and diluted for sequencing according to Illumina sequencing protocol. We aimed to sequence each library to achieve **>**50,000 reads per cell.

After sequencing, the fastQ files were submitted to Cell Ranger version 7.0.0 ‘mkref’, ‘mkfastq’ and ‘count’ functions with a custom genome of *Mus musculus* that contain the viral genome as exons (refdata-gex-mm10-2020-A). The filtered output of ‘counts’ (barcodes.tsv, features.tsv and matrix.mtx) were used for subsequent analysis with Seurat. In Seurat v4, we performed pre-processing of the data (quality controls) and normalization using the SCTransform function for accounting for batch effects. Once all samples were processed, they were integrated into one large Seurat object that contain all the CD45^+^ conditions (two replicates of infected WT CD45^+^ cells, two replicates of infected FcRγ^−/−^ CD45^+^ cells, and one replicate of each mouse genotype as naive controls). We found 18 clusters at a clustering resolution of 0.4. We generated all marker genes of the clusters to assign the cell types within our dataset using the FindAllMarkers function in Seurat. Immune cell subsets were classified by a combination of the top 5 significant genes in each cluster as well as hallmark genes for certain cell types based on the literature (S4A–S4C Fig) [38–62]. For subsequent analysis of the myeloid cells, we subset the corresponding clusters (“0”, “1”, “2”, “3”, “4”, “8”, “16”) into a new Seurat object (18,000 cells in total). We proceeded with re-analysis of this subset, resulting in 9 clusters. Cluster 8, corresponding to B cells, was removed from this analysis of myeloid cells only. As described above, we used FindAllMarkers function to generate all markers genes for characterization of the cell types and subtypes (S6A-S6C Fig). Reads mapping to the viral template were counted and reads per cell were computed in Seurat using the PercentageFeatureSet function, for both the sub-genomic and full-length genome, or aggregated as total viral reads, as shown in Fig 5D. For CD45^-^ cells conditions (two replicates of infected WT and two replicates of infected FcRγ^−/−^ cells) were processed separately as described above and integrated into a larger Seurat object. The viral reads were also assessed in CD45^-^ cells for comparison with CD45^+^ cells (Fig 4D).

### Pathway enrichment and modeling of gene networks

For differentially expressed gene (DEG) analysis, we focused on the myeloid cell subset and the cluster 7 subset (CD8^+^ T cells). Seurat objects, creating an extra column in the metadata that contained the Seurat cluster and the condition these cells came from, then we generated a dataframe containing all possible pairwise comparisons between clusters and conditions. From those, we selected the pairwise comparisons between infected FcRγ^−/−^ to WT (including CD8^+^ T cells that corresponds Cluster 7) or FcRγ^−/−^ Cluster 2 vRNA^+^ to vRNA^-^ for all clusters that had at least a minimum of 50 cells for each condition using the FindMarkers function as described in the differential expression testing vignette in the Seurat documentation. DEGs for comparisons between infected FcRγ^−/−^ to WT clusters (including the myeloid cells subset and CD8^+^ T cells) or FcRγ^−/−^ Cluster 2 vRNA^+^ to vRNA^-^ were imported into Qiagen Ingenuity Pathways Analysis (IPA) (Ingenuity Systems; Qiagen, Redwood City, CA, USA). The list was subjected to a core analysis (Log2FC ≤ -0.58 or ≥0.58, adjusted P value < 0.05), with significant IPA canonical pathways (p value < 0.05) assigned a Z score based on predicted activation state. The graphical summary of the canonical pathways highlights predicted interactions between terms, as curated by IPA.

### Similarity heatmaps of GO terms

Gene ontology analysis was performed on differentially expressed genes (absolute log2-fold change > 1 and FDR-adjusted p-value < 0.1) between infected FcRγ^−/−^ and WT clusters using the clusterProfiler v4.10.1 R package [100]. Significantly enriched gene ontology pathways (FDR-adjusted p-value < 0.001) were then summarized using the simplifyEnrichment v1.12.0 R package [101] to generate similarity heatmaps, revealing distinct sets of gene ontology terms with consistent similarities within each set.

### Statistical analysis

Statistical significance was assigned with P values using GraphPad Prism 9 (La Jolla, CA). Parametric or non-parametric two-tailed statistical tests were selected based on data distribution and whether samples were at the limit of detection of the assay. Specific statistical tests utilized are described in the figure legend of the corresponding data.

## Supporting information

S1 Fig Contralateral foot swelling and viral RNA in tissues of FcRγ−/− mice following MAYV infection. Four-week-old WT or FcRγ^−/−^ C57BL/6N mice were infected subcutaneous in the rear footpad with 10^3^ focus forming units (FFU) of MAYV. (A) Swelling of the contralateral foot was measured prior to infection and for 25 dpi (n = 8 per group, 2 independent experiments). Graphs show mean ± SEM. Statistical significance was determined using a two-way ANOVA with repeated measures and a Sidak’s post-test at each time point. No significant differences were detected at any time point. (B) Indicated tissues were harvested at 3 and 8 dpi and titrated for viral RNA by qRT-PCR with MAYV-specific primers and probe (n = 3 to 12 per group; 2 to 3 independent experiments). Statistical significance was determined by a Mann-Whitney test (**, *P* < 0.01; ****, *P* < 0.0001; ns = not significant). Bars indicate the median value and dotted lines indicate the limit of detection for the assay.
(TIF)

S2 Fig Analysis of the humoral response at post-acute and chronic time points following MAYV infection.
Four-week-old WT or FcRγ^−/−^ C57BL/6N mice were infected subcutaneous in the rear footpad with 10^3^ FFU of MAYV. Serum was collected at indicated time points. (**A**) Avidity of antibodies at 8 dpi was measured using a chaotropic ELISA, with 1:40 diluted serum antibody against recombinant MAYV E2 incubated with either 5M or 3M urea. Serial dilutions of serum were used to determine (**B**) EC_50_ values for IgG or IgM antibodies against captured MAYV virions at 8 dpi or (**C**) IgG subclasses against recombinant E2 at 28 dpi by ELISA (n = 8 to 15 per group; 2 to 3 independent experiments). (**D**) ADCC was determined by luciferase assay using Jurkat effector cells expressing mouse FcγRIV incubated with MAYV infected Vero cells. Statistical significance was determined by a Mann-Whitney test (A-D) *, *P* <0.05; **, *P* < 0.01; ***, *P* < 0.001; ****, *P* < 0.0001; ns = not significant.
(TIF)

S3 Fig Flow gating scheme and adaptive immune responses. (**A**) Flow gating scheme for identification of immune cell subsets. (**B**-**C**) Four-week-old WT or FcRγ^−/−^ C57BL/6N mice were infected subcutaneous in the rear footpad with 10^3^ FFU of MAYV. Single cell suspensions were isolated from the ipsilateral foot and proximal skin at (**B**) 3, 8, and 10 dpi or (**C**) 28 dpi stained for immune cells (CD45^+^), CD4 T cells (CD3^+^CD4^+^), CD8 T cells (CD3^+^CD8^+^), and B cells (CD3^-^CD19^+^) and analyzed by flow cytometry to determine the total numbers of viable cells or percentage of CD45^+^ cells (n = 5 to 8 per group; 3 independent experiments). (**C**) The gray bar represents the range of total cells and percentage of CD45^+^ cells from WT and FcRγ^−/−^ naïve mice. Graphs show mean ± SEM. Statistical significance was determined using a Mann-Whitney test at individual time points. *, *P* <0.05; **, *P* < 0.01; ***, *P* < 0.001; ns = not significant.
(TIF)

S4 Fig Classification of immune cell clusters by genetic signature. (**A**) Dot plot of the top 5 most significant genes in each cluster from integrated RNA sequencing data, indicating log_2_FC and proportion of cells expressing each gene. (**B**) Cell identification of clusters based on additional key genes. (**C**) Dot plot of additional key gene identifiers in (B) showing log_2_FC and proportion of cells expressing each gene. (**D**) Distribution of cells across each cluster, shown for each individual mouse, indicating the log_2_(enrichment) of the clusters between the groups [n = 2 per infected condition, n = 1 for WT naive control, and n = 1 for FcRγ^−/−^ (KO) naive control]. Enrichment of B cells (Cluster 6) in the FcRγ^−/−^ naive sample is believed to be caused by a microbreak during initial tissue harvest, which is not present in any of the other samples. (**E**) MAYV RNA was quantified from CD45^-^ cells sorted from the ipsilateral foot at 10 dpi by RT-qPCR with E2 specific primers and probe. Statistical significance was determined by Mann-Whitney test, with the differences between the groups being non-significant.
(TIF)

S5 Fig Overview of enriched pathways in FcRγ−/− CD8+ T cells.
(**A**) Volcano plots showing the average fold change (log2) and adjusted p value in the comparisons between FcRγ^−/−^ and WT CD8^+^ T cells from cluster 7. The condition-specific expression indicates the fold change (log2) in the cells that have the gene detectable. (**B**) Enriched canonical pathways by IPA between FcRγ^−/−^ cluster 7 compared to WT mice. Orange dots indicate a positive z-score, blue dots indicate a negative z-score, white dots represent a z-score of 0, and a z-score could not be defined in gray dots. (**C**) Differentially expressed genes (DEGs) enriched in FcRγ^−/−^ mice for cluster 7 analyzed using GO term analysis. Significant ontology terms were clustered based on semantic similarity of member gene sets using simplifyEnrichment and hand annotated based on biological theme.
(TIF)

S6 Fig Classification of myeloid subclusters by genetic signature.
(**A**) Dot plot of the top 5 most significant genes in the myeloid subcluster analysis, indicating log_2_FC and proportion of cells expressing each gene. (**B**) Additional key genes for cell identification of myeloid subcluster based on expert curation. (**C**) Dot plot of additional key gene identifiers in (B) showing log_2_FC and proportion of cells expressing each gene. (**D**) Distribution of cells across the subclusters, shown for each individual mouse, indicating the log_2_(enrichment) of the subclusters between the groups (n = 2 per infected condition, n = 1 for WT naive control, and n = 1 for FcRγ^−/−^ (KO) naive control). (**E**) The proportion of cells for each subcluster, separated by genotype and the presence of MAYV RNA, showing the log_2_(enrichment) of each subcluster between viral RNA positive and negative cells. (n = 2 per infected condition, n = 1 for WT naive control, and n = 1 for FcRγ^−/−^ (KO) naive control).
(TIF)

S7 Fig Overview of enriched pathways in FcRγ−/− subclusters.
(**A**) Differentially expressed genes (DEGs) enriched in FcRγ^−/−^ mice for each subcluster were analyzed using GO term analysis. Significant ontology terms were clustered based on semantic similarity of member gene sets using simplifyEnrichment and hand annotated based on biological theme. (**B**) Volcano plots showing the average fold change (log2) and adjusted p value in the comparisons between FcRγ^−/−^ vRNA^+^ and vRNA^-^ cells from cluster 2. The condition-specific expression indicates the fold change (log2) in the cells that have the gene detectable. (**C**) GO term analysis of DEGs between FcRγ^−/−^ vRNA^+^ and vRNA^-^ cells from cluster 2. Significant ontology terms were clustered based on semantic similarity of member gene sets using simplifyEnrichment and hand annotated based on biological theme.
(TIF)

S1 Data Data underlying the findings in the manuscript.
(XLSX)
