# Supplementary figures and images for: Interaction of the endogenous antibody response with activating FcγRs enhance control of Mayaro virus through monocytes
Source: PLoS Pathog. 2025 Feb 24;21(2):e1012944. doi: 10.1371/journal.ppat.1012944 (PMC11884725; doi:10.1371/journal.ppat.1012944)

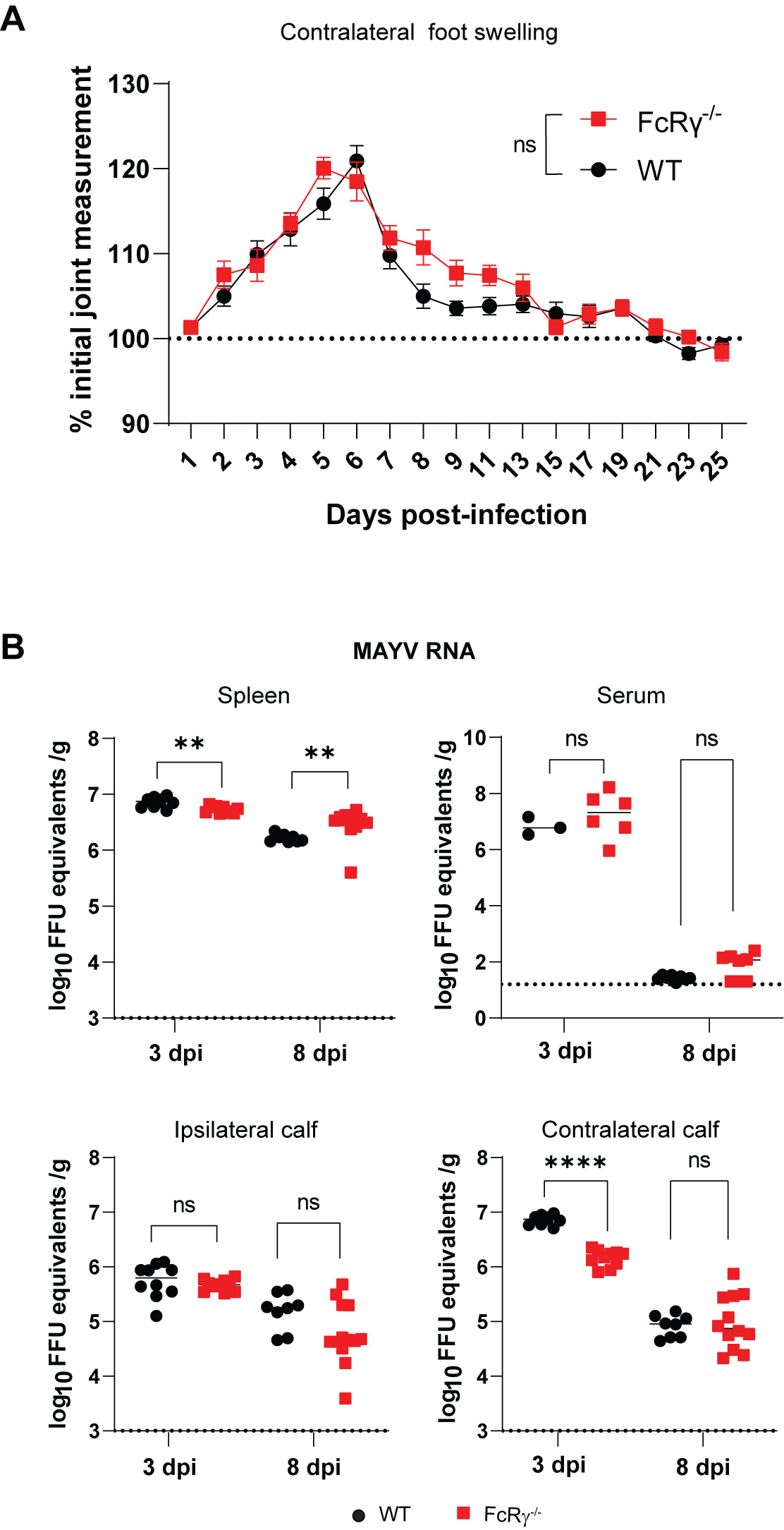

Supplement: S1 Fig — Four-week-old WT or FcRγ−/− C57BL/6N mice were infected subcutaneous in the rear footpad with 103 focus forming units (FFU) of MAYV. (A) Swelling of the contralateral foot was measured prior to infection and for 25 dpi (n = 8 per group, 2 independent experiments). Graphs show mean ± SEM. Statistical significance was determined using a two-way ANOVA with repeated measures and a Sidak’s post-test at each time point. No significant differences were detected at any time point. (B) Indicated tissues were harvested at 3 and 8 dpi and titrated for viral RNA by qRT-PCR with MAYV-specific primers and probe (n = 3 to 12 per group; 2 to 3 independent experiments). Statistical significance was determined by a Mann-Whitney test (**, P < 0.01; ****, P < 0.0001; ns = not significant). Bars indicate the median value and dotted lines indicate the limit of detection for the assay. (TIF) [file ppat.1012944.s001.tif]

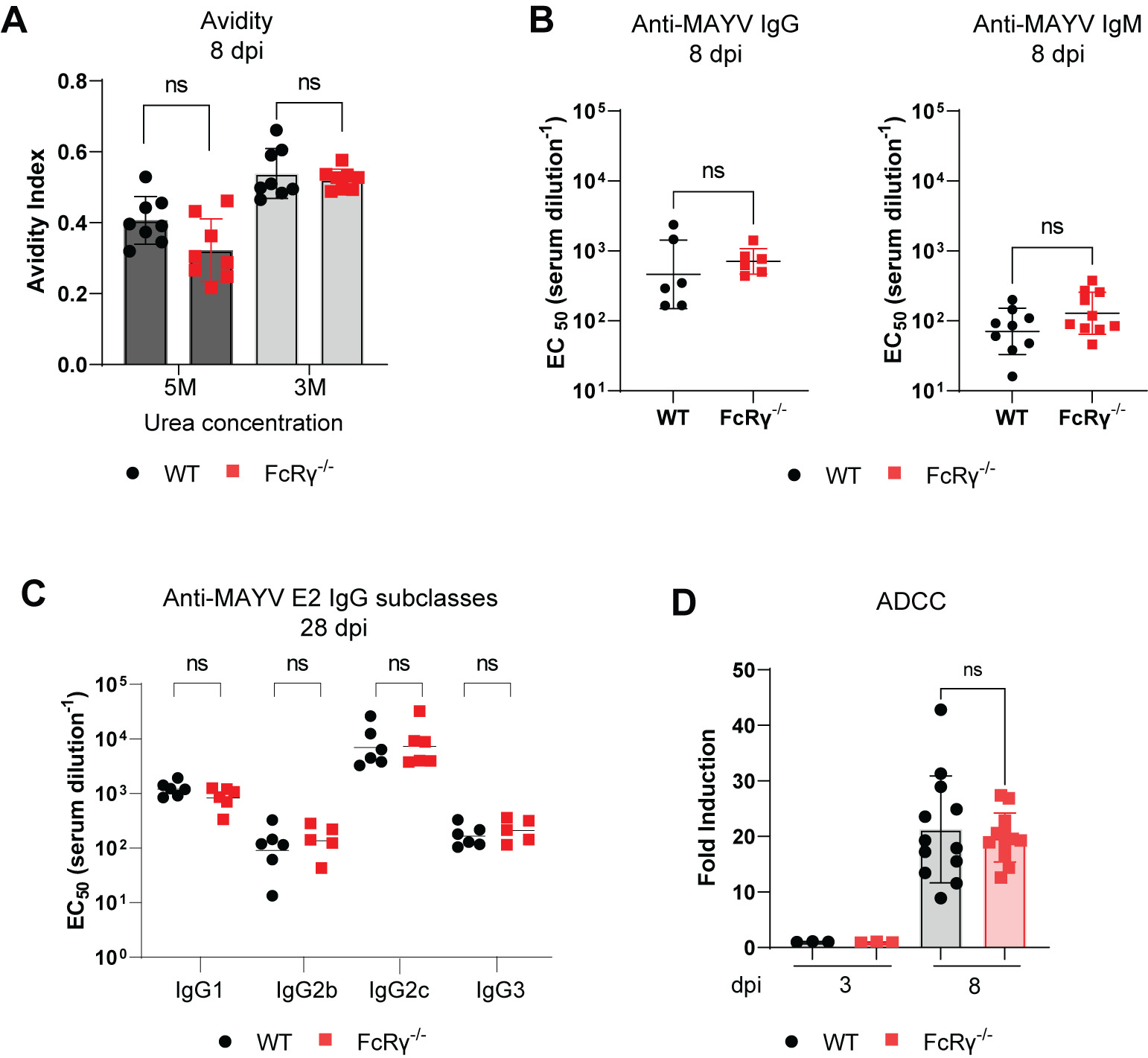

Supplement: S2 Fig — Four-week-old WT or FcRγ−/− C57BL/6N mice were infected subcutaneous in the rear footpad with 103 FFU of MAYV. Serum was collected at indicated time points. (A) Avidity of antibodies at 8 dpi was measured using a chaotropic ELISA, with 1:40 diluted serum antibody against recombinant MAYV E2 incubated with either 5M or 3M urea. Serial dilutions of serum were used to determine (B) EC50 values for IgG or IgM antibodies against captured MAYV virions at 8 dpi or (C) IgG subclasses against recombinant E2 at 28 dpi by ELISA (n = 8 to 15 per group; 2 to 3 independent experiments). (D) ADCC was determined by luciferase assay using Jurkat effector cells expressing mouse FcγRIV incubated with MAYV infected Vero cells. Statistical significance was determined by a Mann-Whitney test (A-D) *, P <0.05; **, P < 0.01; ***, P < 0.001; ****, P < 0.0001; ns = not significant. (TIF) [file ppat.1012944.s002.tif]

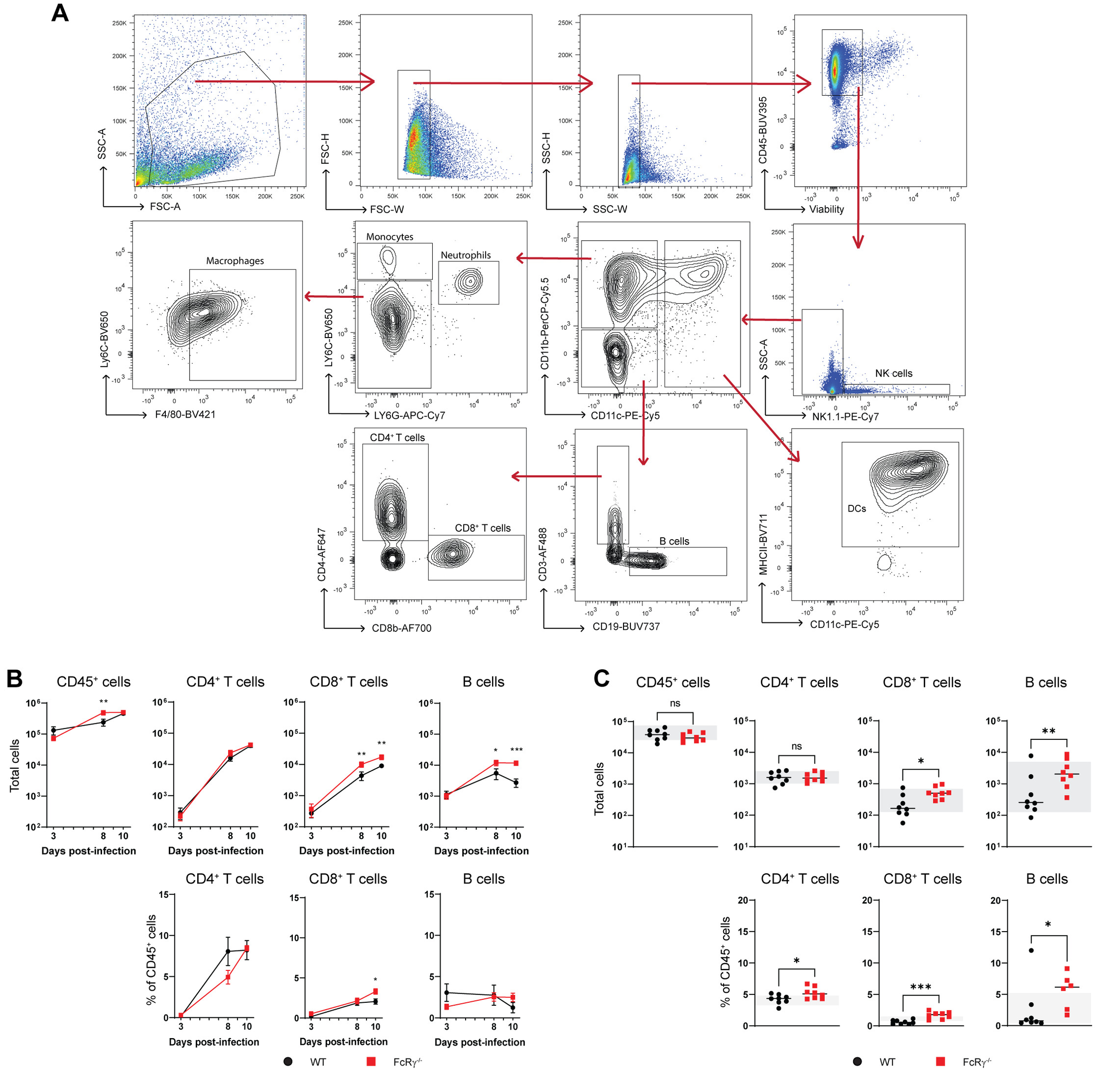

Supplement: S3 Fig — (A) Flow gating scheme for identification of immune cell subsets. (B-C) Four-week-old WT or FcRγ−/− C57BL/6N mice were infected subcutaneous in the rear footpad with 103 FFU of MAYV. Single cell suspensions were isolated from the ipsilateral foot and proximal skin at (B) 3, 8, and 10 dpi or (C) 28 dpi stained for immune cells (CD45+), CD4 T cells (CD3+CD4+), CD8 T cells (CD3+CD8+), and B cells (CD3-CD19+) and analyzed by flow cytometry to determine the total numbers of viable cells or percentage of CD45+ cells (n = 5 to 8 per group; 3 independent experiments). (C) The gray bar represents the range of total cells and percentage of CD45+ cells from WT and FcRγ−/− naïve mice. Graphs show mean ± SEM. Statistical significance was determined using a Mann-Whitney test at individual time points. *, P <0.05; **, P < 0.01; ***, P < 0.001; ns = not significant. (TIF) [file ppat.1012944.s003.tif]

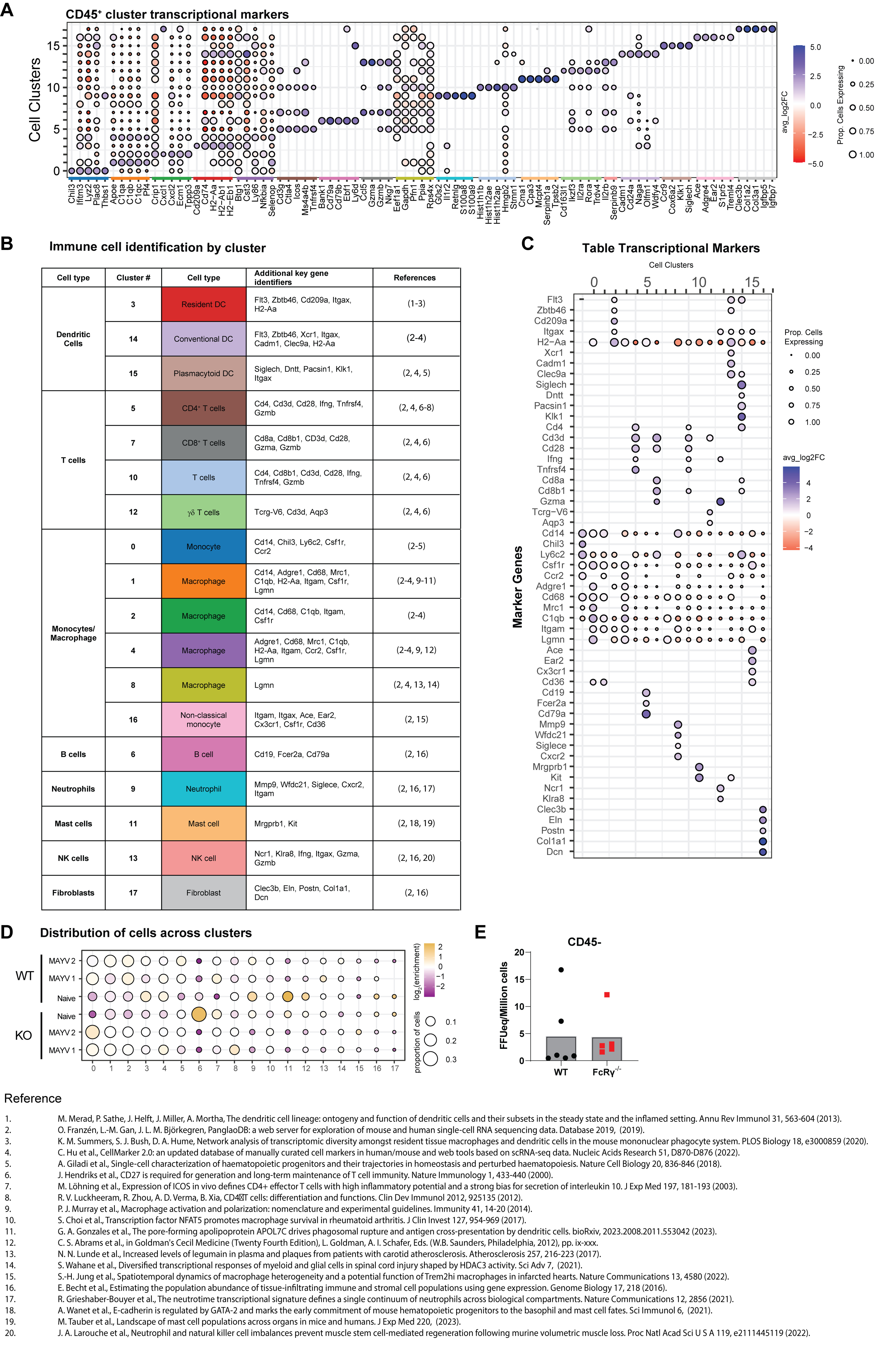

Supplement: S4 Fig — (A) Dot plot of the top 5 most significant genes in each cluster from integrated RNA sequencing data, indicating log2FC and proportion of cells expressing each gene. (B) Cell identification of clusters based on additional key genes. (C) Dot plot of additional key gene identifiers in (B) showing log2FC and proportion of cells expressing each gene. (D) Distribution of cells across each cluster, shown for each individual mouse, indicating the log2(enrichment) of the clusters between the groups [n = 2 per infected condition, n = 1 for WT naive control, and n = 1 for FcRγ−/− (KO) naive control]. Enrichment of B cells (Cluster 6) in the FcRγ−/− naive sample is believed to be caused by a microbreak during initial tissue harvest, which is not present in any of the other samples. (E) MAYV RNA was quantified from CD45- cells sorted from the ipsilateral foot at 10 dpi by RT-qPCR with E2 specific primers and probe. Statistical significance was determined by Mann-Whitney test, with the differences between the groups being non-significant. (TIF) [file ppat.1012944.s004.tif]

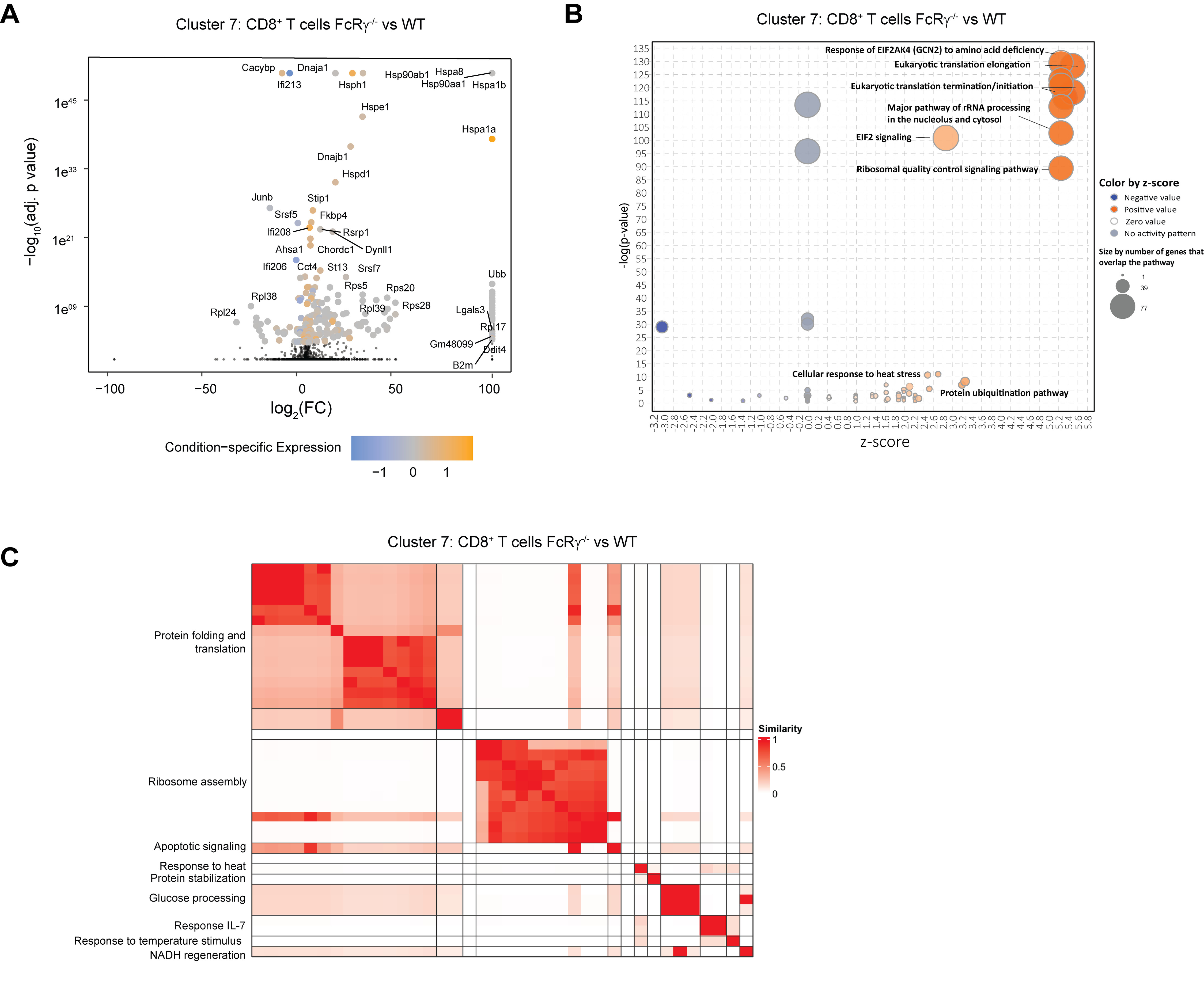

Supplement: S5 Fig — (A) Volcano plots showing the average fold change (log2) and adjusted p value in the comparisons between FcRγ−/− and WT CD8+ T cells from cluster 7. The condition-specific expression indicates the fold change (log2) in the cells that have the gene detectable. (B) Enriched canonical pathways by IPA between FcRγ−/− cluster 7 compared to WT mice. Orange dots indicate a positive z-score, blue dots indicate a negative z-score, white dots represent a z-score of 0, and a z-score could not be defined in gray dots. (C) Differentially expressed genes (DEGs) enriched in FcRγ−/− mice for cluster 7 analyzed using GO term analysis. Significant ontology terms were clustered based on semantic similarity of member gene sets using simplifyEnrichment and hand annotated based on biological theme. (TIF) [file ppat.1012944.s005.tif]

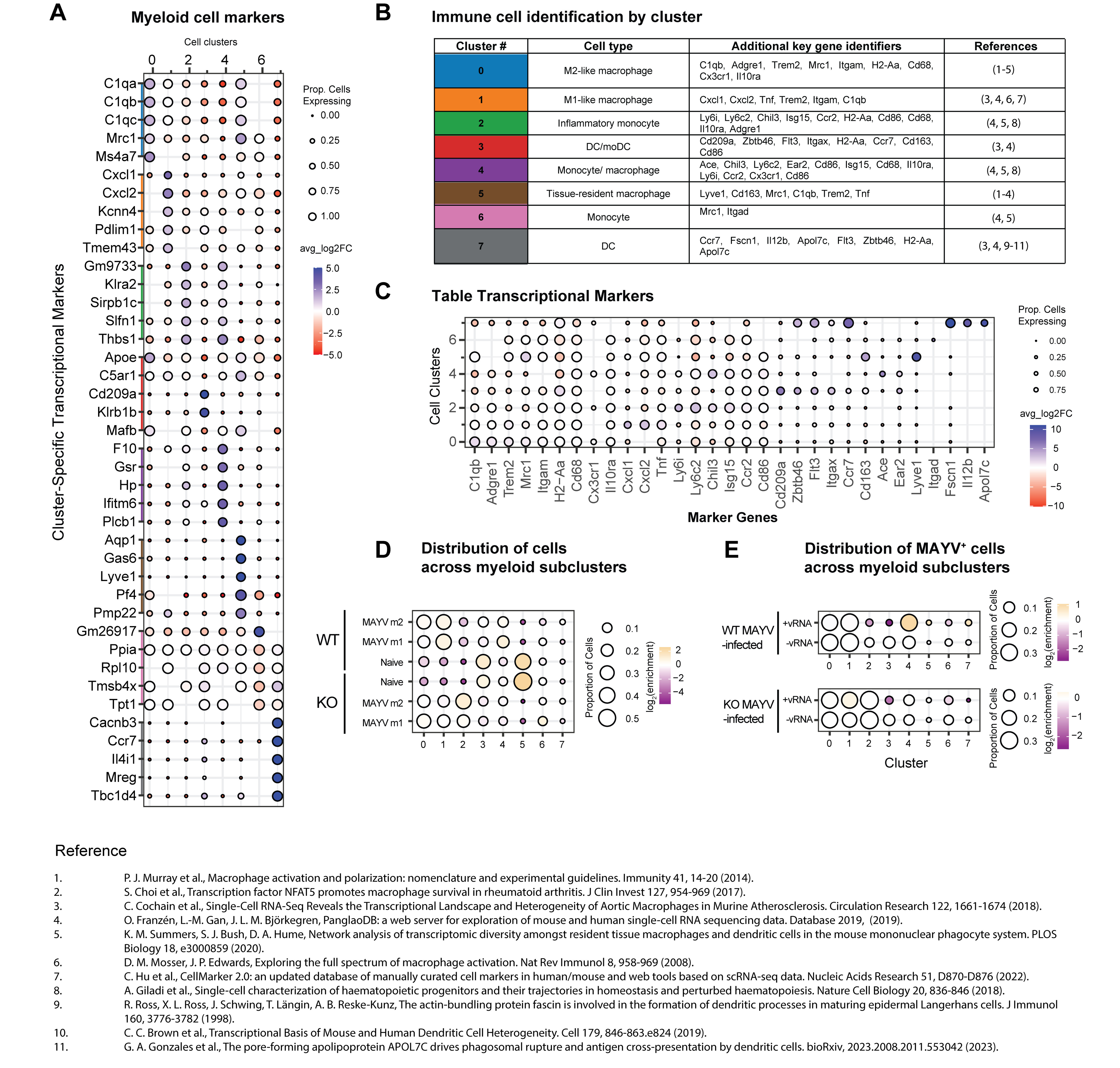

Supplement: S6 Fig — (A) Dot plot of the top 5 most significant genes in the myeloid subcluster analysis, indicating log2FC and proportion of cells expressing each gene. (B) Additional key genes for cell identification of myeloid subcluster based on expert curation. (C) Dot plot of additional key gene identifiers in (B) showing log2FC and proportion of cells expressing each gene. (D) Distribution of cells across the subclusters, shown for each individual mouse, indicating the log2(enrichment) of the subclusters between the groups (n = 2 per infected condition, n = 1 for WT naive control, and n = 1 for FcRγ−/− (KO) naive control). (E) The proportion of cells for each subcluster, separated by genotype and the presence of MAYV RNA, showing the log2(enrichment) of each subcluster between viral RNA positive and negative cells. (n = 2 per infected condition, n = 1 for WT naive control, and n = 1 for FcRγ−/− (KO) naive control). (TIF) [file ppat.1012944.s006.tif]

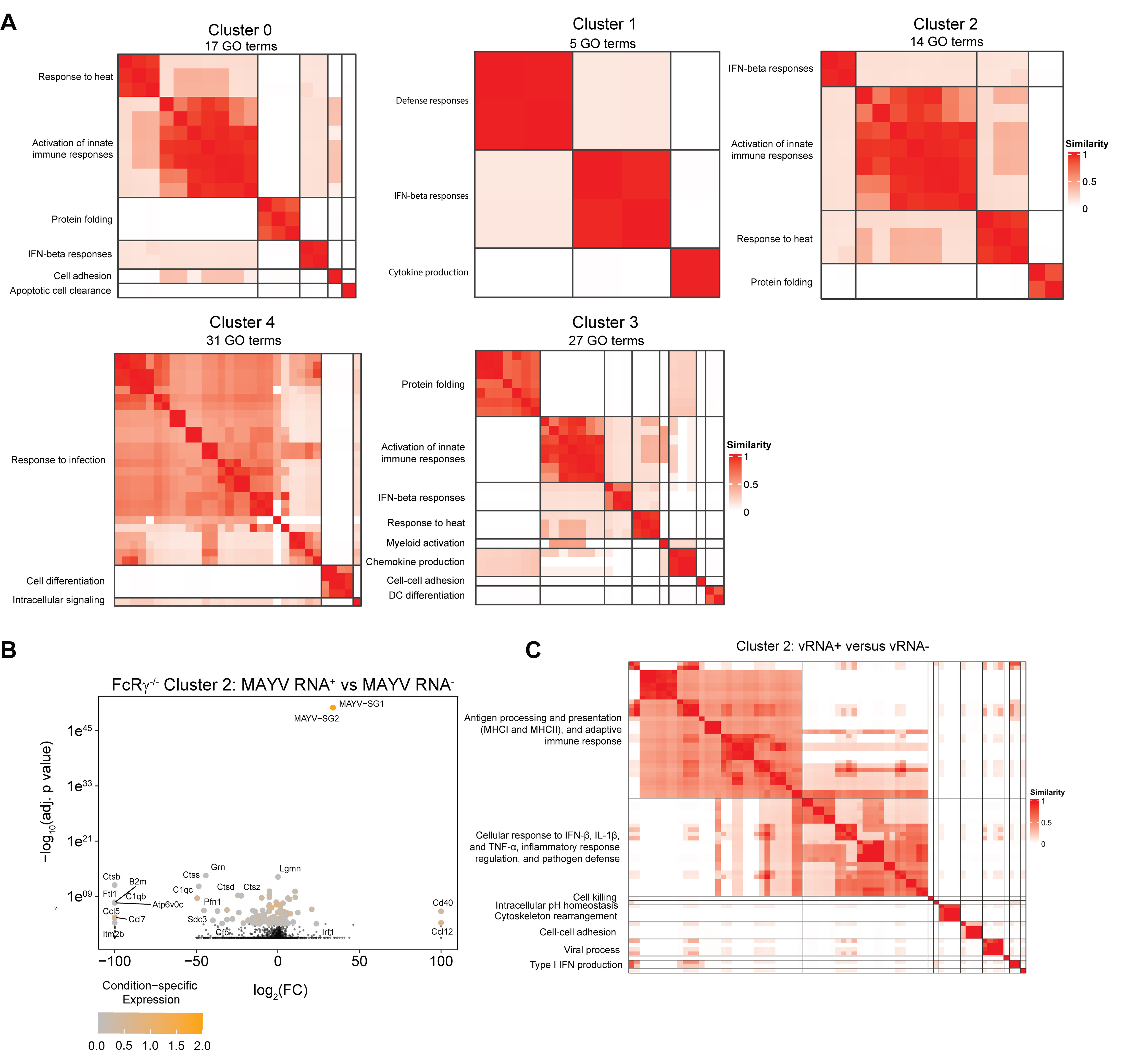

Supplement: S7 Fig — (A) Differentially expressed genes (DEGs) enriched in FcRγ−/− mice for each subcluster were analyzed using GO term analysis. Significant ontology terms were clustered based on semantic similarity of member gene sets using simplifyEnrichment and hand annotated based on biological theme. (B) Volcano plots showing the average fold change (log2) and adjusted p value in the comparisons between FcRγ−/− vRNA+ and vRNA- cells from cluster 2. The condition-specific expression indicates the fold change (log2) in the cells that have the gene detectable. (C) GO term analysis of DEGs between FcRγ−/− vRNA+ and vRNA- cells from cluster 2. Significant ontology terms were clustered based on semantic similarity of member gene sets using simplifyEnrichment and hand annotated based on biological theme. (TIF) [file ppat.1012944.s007.tif]
